# Bacterial terpene biosynthesis: challenges and opportunities for pathway engineering

**DOI:** 10.3762/bjoc.15.283

**Published:** 2019-11-29

**Authors:** Eric J N Helfrich, Geng-Min Lin, Christopher A Voigt, Jon Clardy

**Affiliations:** 1 Harvard Medical School, Department of Biological Chemistry and Molecular Pharmacology, Boston, United States; 2 Massachusetts Institute of Technology, Department of Biological Engineering, Cambridge, United States

**Keywords:** bacterial sesquiterpenes and diterpenes, cytochrome P450, pathway engineering, synthetic biology, terpene biosynthesis, terpene cyclase

## Abstract

Terpenoids are the largest and structurally most diverse class of natural products. They possess potent and specific biological activity in multiple assays and against diseases, including cancer and malaria as notable examples. Although the number of characterized terpenoid molecules is huge, our knowledge of how they are biosynthesized is limited, particularly when compared to the well-studied thiotemplate assembly lines. Bacteria have only recently been recognized as having the genetic potential to biosynthesize a large number of complex terpenoids, but our current ability to associate genetic potential with molecular structure is severely restricted. The canonical terpene biosynthetic pathway uses a single enzyme to form a cyclized hydrocarbon backbone followed by modifications with a suite of tailoring enzymes that can generate dozens of different products from a single backbone. This functional promiscuity of terpene biosynthetic pathways renders terpene biosynthesis susceptible to rational pathway engineering using the latest developments in the field of synthetic biology. These engineered pathways will not only facilitate the rational creation of both known and novel terpenoids, their development will deepen our understanding of a significant branch of biosynthesis. The biosynthetic insights gained will likely empower a greater degree of engineering proficiency for non-natural terpene biosynthetic pathways and pave the way towards the biotechnological production of high value terpenoids.

## Introduction

**Figure 1:**
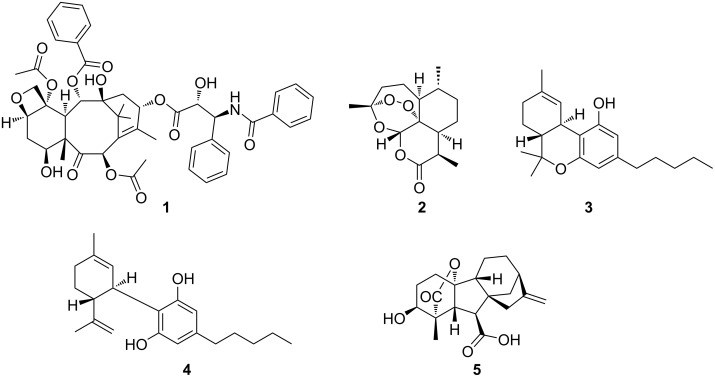
Examples of bioactive terpenoids.

Evolutionary diversification of terpene biosynthetic pathways has resulted in the largest and most structurally diverse class of specialized metabolites on the planet. To date more than 70,000 terpenoids (dictionary of natural products) have been characterized and grouped into more than 400 structural families – the vast majority of which have been isolated from plants and fungi [1]. Their structural diversity reflects the breadth of their functional roles, which range from widely distributed metabolites like cholesterol, to those with more restricted distribution like vitamins A and D, carotenoids, and steroid hormones, to some with highly restricted distribution like pheromones, fragrances, and defense metabolites [2–3]. Many of the more restricted members possess significant biological activities, like the anticancer agent taxol (**1**) [4] or the antimalarial agent artemisinin (**2**, Figure 1) [5–6]. The structural diversity and functional utility of this class of specialized metabolites have combined to encourage efforts to apply the tools of synthetic biology to engineer pathways that will expand molecular diversity, especially around scaffolds associated with high-value compounds.

The biosynthetic logic of terpene formation differs significantly from the logic employed by other classes of secondary metabolite biosynthetic pathways. Bacterial thiotemplated assembly lines, such as type I polyketide synthases (PKS) and nonribosomal peptide synthetases (NRPS), are modular, with each module contributing a distinct fragment to the final product’s core structure – a short-chain carboxylic acid (PKS) or an amino acid (NRPS). The modularly defined template can be further modified by tailoring enzymes, but the core structure can be inferred from the organization of the biosynthetic genes and the modular architecture of the associated proteins [7–8].

Terpene biosynthesis has a very different logic. Five-carbon units called isoprenes are joined to create a linear polyene with branching methyl groups that form the core hydrocarbon structure in a single enzyme-catalyzed step [9]. The enzyme, which is called terpene cyclase, holds the linear methyl-branched polyene in a defined conformation that initiates a series of carbocation-driven cyclizations and rearrangements, creating the basic hydrocarbon skeleton of a terpene [10–11]. This basic hydrocarbon skeleton is then modified to generate a large number of terpenoid structures, which can be further modified by addition of other building blocks, like sugars, amino acids, or fatty acids [12]. Terpenes are named for the number of five-carbon units that form their hydrocarbon skeletons. Our review focuses on sesqui- (C_15_) and diterpenes (C_20_) because these subgroups, after undergoing extensive oxidative modification, have molecular characteristics that are most similar to those of known drugs. Emphasis will be placed on terpene pathways from bacteria, as their biosynthetic pathways usually have the genes encoding the terpene cyclase and modifying enzyme in close proximity, which simplifies both analysis and pathway engineering. The review will begin with a brief description of terpene families with a special focus on a few illustrative examples with pharmaceutical significance that highlight general hallmarks of terpene biosynthesis, then describe the natural biosynthetic pathways in more detail before moving on to describing the progress, promise, and obstacles to engineering terpene biosynthetic pathways.

## Review

### Remaining challenges in terpene (bio)synthesis

While the large number of characterized terpenes and their biological and medical significance would suggest that there are good tools for terpene synthesis along with a good understanding of terpene biosynthesis, significant gaps in both remain. Taxol (**1**), a plant-derived terpenoid, provides an illustrative example. Taxol was structurally characterized in 1971 [4] and approved by the FDA as an anticancer agent in 1992 [13]. Today, almost 50 years after its initial report, despite its blockbuster status in cancer therapy [14] and multiple research efforts, there is no sustainable synthetic or biosynthetic approach to this molecule. More than half a dozen total syntheses, each requiring 37 steps [15–16] or more [14], have been reported, yielding at best 32 mg of the drug in toto*,* while the yearly pharmaceutical requirements are greater than 10^9^ mg [17]. The cyclase that forms the hydrocarbon framework has been known for more than 20 years [18–19], but the precise biosynthetic modifications leading to the final molecule are still not fully understood [20]. These shortcomings were successfully addressed in a practical sense through semi-synthesis from biotechnologically produced taxol precursors [21], but a completely engineered pathway could provide a more efficient solution to taxol and specific analogs.

There are additional puzzles about terpene biosynthesis that engineered pathways could address. As many other terpenes, **1** is produced along with a large suite of related molecules, the taxanes [17]. This pathway promiscuity can be illustrated with another popular example of a terpene family: the cannabinoids, with more than 100 members used for both medical and recreational purposes. While its most prominent member (−)-*trans*-Δ⁹-tetrahydrocannabinol, better known as THC (**3**, Figure 1), is widely known as the principal psychoactive constituent of cannabis, cannabidiol (CBD, **4**, Figure 1) for example, shows strongly diminished psychoactive properties but has promising anti-inflammatory [22], antischizophrenic [23], and anti-epileptic bioactivities [24–25]. Modifying the cannabinoid profile through engineering would therefore be of significant interest.

The biosynthetic promiscuity, the hallmark of terpene biosynthesis, sets terpenes apart from other natural product classes and is a product of their distinctive biosynthetic logic. Biosynthetic core enzymes of well-characterized classes of natural products, such as modular thiotemplate assembly lines (NRPSs, PKSs), are usually highly specific and produce only a few closely related natural product analogs. Adenylation domains in NRPSs [26–28], acyltransferase (AT) domains in *cis*-AT PKSs [29], and ketosynthase domains in *trans*-AT PKSs [30–31] are highly substrate-specific and function as gatekeepers to ensure that a single natural product is produced with high efficiency. Erroneous or stalled intermediates are removed from the assembly lines [32–33]. Novelty in these assembly lines is achieved through the recombination of module series (*trans*-AT PKSs) [34], the exchange of large subdomains (NRPSs) [35], and the duplication followed by diversification of entire modules (NRPSs and *cis*-AT PKSs) [36]. As a result, dysfunctionality is a constant threat for a metabolic pathway during evolution.

**Figure 2:**
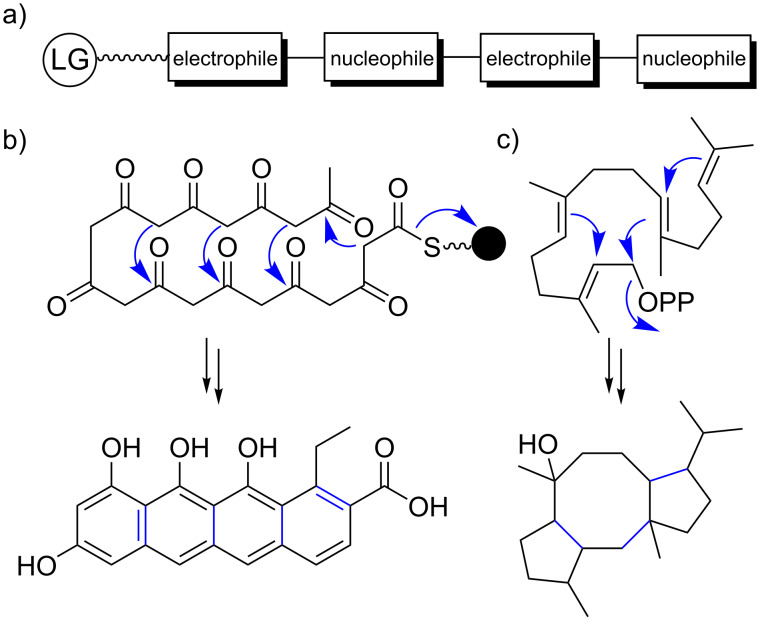
Repetitive electrophilic and nucleophilic functionalities in terpene and type II PKS-derived polyketide biosynthesis. a) Schematic representation. b) Type II PKS-derived polyketide biosynthesis. c) Terpene biosynthesis. LG: leaving group, black ball: acyl carrier protein.

In contrast, the underlying mechanistic logic of terpene biosynthesis is based on repetitive electrophilic and nucleophilic functionalities in each oligomeric substrate, similar to nonmodular type II PKSs, coupled with conformational flexibility for enzyme-mediated juxtaposition of complementary pairs of these functionalities (Figure 2) [9]. It is this intrinsic and repetitive reactivity that can be easily tuned by natural selection [9]. As a result, a single terpene cyclase (TC) can produce dozens of hydrocarbon scaffolds that can differ significantly from each other [9,37]. This biosynthetic promiscuity should be regarded as a valuable feature rather than a bug in the system. According to the “screening hypothesis" [38–39] (more recently and appropriately also referred to as the “diversity-based hypothesis" [40]), potent biological activity is a rare property of any molecule in nature [39]. Therefore, evolution would likely favor organisms that can generate and retain chemical diversity at a low cost [38]. As a result, producing and “screening” a large number of specialized metabolites for potent TCs that can generate multiple products from simple building blocks is a huge evolutionary advantage; even more so when combined with tailoring enzymes that have a broad substrate tolerance and catalyze multiple, sequential tailoring reactions [40]. This diversification process resembles the strategy embedded in the construction of combinatorial libraries by organic chemists to generate chemical diversity. The biosynthetic promiscuity is not a result of intrinsic "sloppiness" of terpenoid biosynthetic pathways, as terpenes that are classified as primary metabolites, such as cholesterol, are produced with high fidelity [40]. In contrast, more promiscuous terpene pathways might only be a few mutations away from novel highly bioactive natural products that might meet new selective needs [40]. All it takes is the ability to generate a small amount of terpene side product that nature can utilize as a starting point to reinforce and refine the pathway [9,40]. As such, terpene pathways allow for quantitative control of product outcomes, while PKS and NRPS pathways feature qualitative control [40]. The gibberellins (e.g., gibberellin A4 (**5**), Figure 1) are an extreme example illustrating the promiscuity of terpene biosynthetic pathways with more than 130 different family members reported [40]. In fact, different pathways have evolved in plants, fungi, and bacteria for this fascinating compound family in an extreme case of convergent evolution [41–42]. While the plant and fungal biosynthetic pathways are well studied [42], the bacterial pathway was studied to a lesser degree until recently [41] – something that can be largely attributed to the historic perception that bacteria are not capable of producing complex terpenoids.

**Figure 3:**
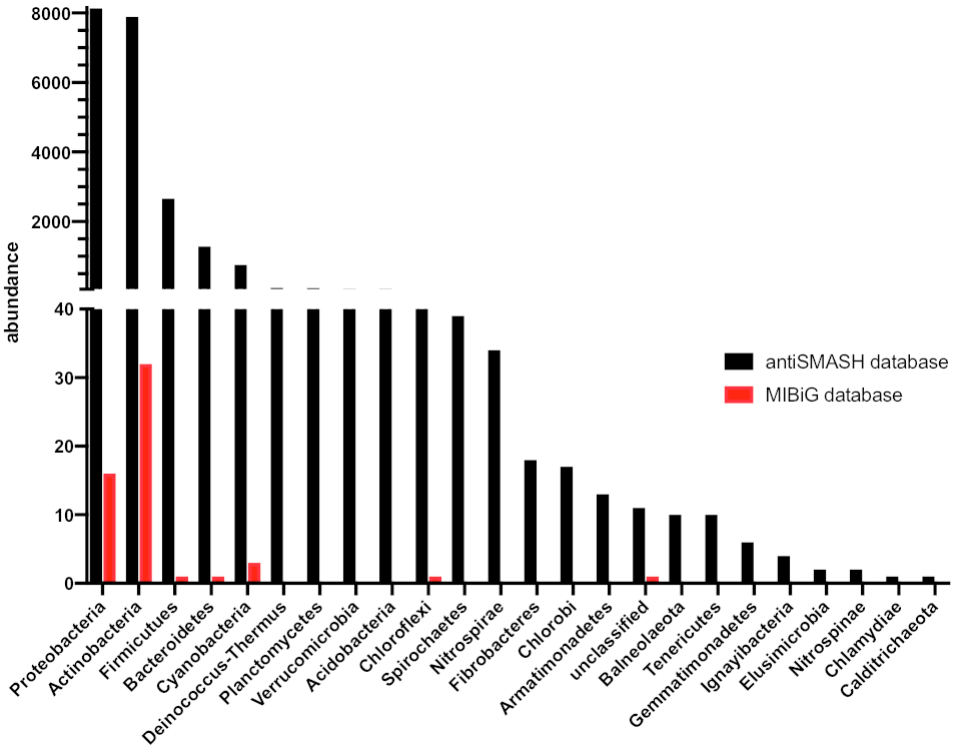
Abundance and distribution of bacterial terpene biosynthetic gene clusters as determined by genome mining (black) compared to experimentally characterized bacterial terpene BGCs (red).

The historic neglect of bacterial terpenes becomes most apparent when the literature is mined for characterized bacterial terpene biosynthetic gene clusters (BGCs). While the antiSMASH database (online repository of BGCs predicted by the genome mining platform antiSMASH) lists more than 4,000 bacterial terpene BGCs [43], only 127 have been characterized and deposited in the MIBiG database (repository of characterized BGCs) to date (Figure 3) [44].

The comparatively small number of characterized bacterial terpenes can likely be attributed to three factors: 1) the early misconception that bacteria are not capable of producing complex terpenoids, 2) the lack of genome mining platforms for terpene biosynthetic gene clusters and the inability to perform rational structure predictions based on genome sequence information, and finally 3) low production titers of bacterial terpenoids under standard laboratory conditions. These factors, in combination with general terpene properties such as lack of UV-absorbing functional groups, poor ionization properties, and ubiquitous odiferous terpenes that overshadow characteristic terpene signals (branching methyl groups) in NMR experiments, render the targeted isolation of terpenes highly challenging. Therefore, heterologous expression in modified host organisms could be the method of choice in most studies.

### Understanding the mechanistic logic of terpene biosynthesis

**Figure 4:**
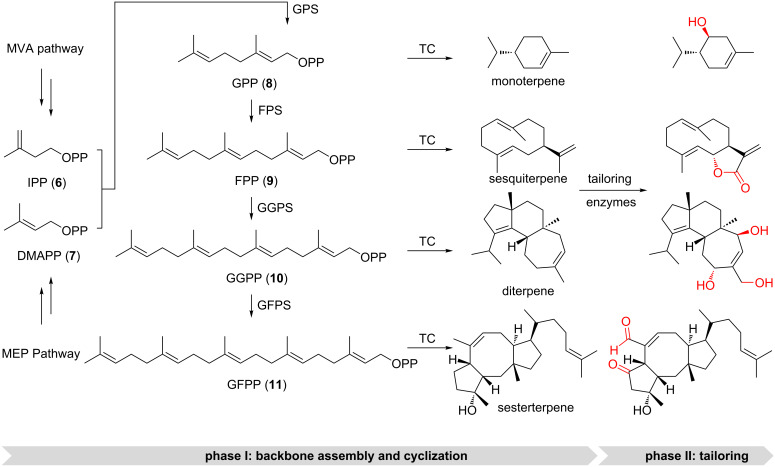
Terpenoid biosynthesis. Terpenoid biosynthesis is divided into two phases, 1) terpene scaffold generation and 2) terpene scaffold functionalization. Representative examples are shown with the modifications installed in the tailoring step highlighted in red. MEP: 2-*C*-methyl-ᴅ-erythritol 4-phosphate pathway, MVA: mevalonic acid pathway, IPP: isopentenyl pyrophosphate, DMAPP: dimethylallyl pyrophosphate, GPS: geranyl pyrophosphate synthase, GPP: geranyl pyrophosphate, FPS: farnesyl pyrophosphate synthase, FPP: farnesyl pyrophosphate, GGPS: geranylgeranyl pyrophosphate synthase, GGPP: geranylgeranyl pyrophosphate, GFPS: geranylfarnesyl pyrophosphate synthase, and GFPP geranylfarnesyl pyrophosphate.

The entry points of terpene biosynthesis are isopentenyl pyrophosphate (IPP, **6**) and dimethylallyl pyrophosphate (DMAPP, **7**), which are assembled through the 2-*C*-methyl-ᴅ-erythritol 4-phosphate (MEP) or the mevalonic acid (MVA) pathway. Each pathway uses different precursors and enzymes, and different organisms utilize either or both pathways. A typical textbook description then divides terpene biosynthesis into two phases (Figure 4): 1) hydrocarbon backbone assembly and cyclization catalyzed by oligoprenyl synthetases and terpene cyclases (terpene synthases, TCs), respectively, to generate terpene scaffolds and 2) the decoration phase, during which the terpene scaffold can get heavily modified by tailoring enzymes [45–47].

### Phase 1) terpene scaffold generation

Unlike core biosynthetic enzymes or domains of other natural product classes (PKSs and NRPSs), bacterial TCs show only little overall sequence similarity [1]. The lack of conservation in primary sequence has slowed down our understanding of terpene cyclization and, as a result, hampered the development of efficient genome mining platforms for the detection of bacterial terpene BGCs. antiSMASH [48] is the only open source web application that annotates bacterial terpene BGCs. As a result of the low conserved sequence similarity between TCs, terpene BGCs are missed more frequently by antiSMASH than other BGC classes [49].

In contrast to the well-characterized biosynthetic rules of thiotemplate biosynthetic pathways that enable relatively precise predictions of natural product core structures (e.g., the colinearity rule in NRPSs and *cis*-acyltransferase polyketide synthases [8]), no such rules exist for the predictions of the cyclic hydrocarbon backbone produced by TCs [1]. This is likely a result of the inherent differences between these biosynthetic machineries. In modular assembly lines, each individual domain is responsible for one defined biosynthetic transformation, while TCs rather act as chaperones [10] to guide oligoprenyl pyrophosphate precursors through a cascade of cyclization reactions.

**Figure 5:**
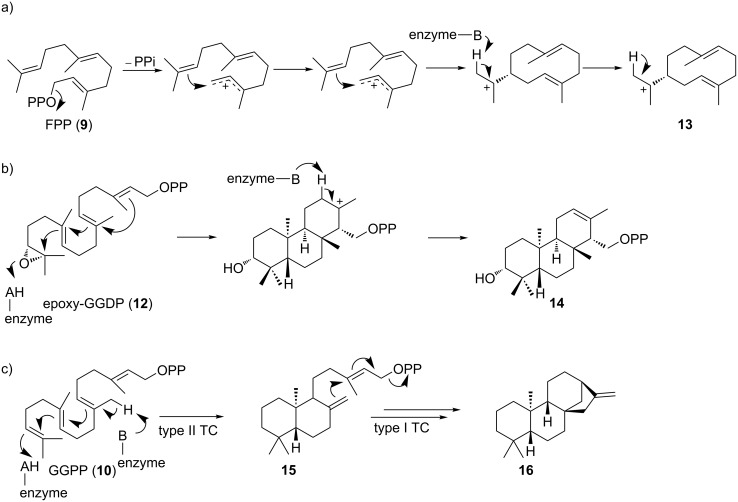
Mechanisms for type I, type II, and type II/type I tandem terpene cyclases. a) Tail-to-head class I germacrene A (**13**) cyclase. b) Head-to-tail type II brasilicardin (**14**) cyclase. c) Type II ent-copalyl diphosphate (**15**) synthase and type I ent-kaurene (**16**) cyclase.

Textbook TCs are categorized into two classes and differ in their mechanisms of substrate activation as well as their protein folds [37,50–52]. Type I TCs trigger the formation of highly reactive allylic cations by heterolytic cleavage of the terminal pyrophosphate of farnesyl diphosphate (FPP, **9**) or geranylgeranyl diphosphate (GGPP, **10**, Figure 5a) [11,53]. Upon binding of the precursor, the TC undergoes conformational changes to seal the hydrophobic binding pocket [54]. This induced-fit mechanism locks the acyclic precursor into a defined, preorganized conformation that positions the leaving pyrophosphate group and the nucleophilic alkenes in proximity to initiate the C–C-bond forming, carbocation-mediated cascade reactions [10]. The hydrophobic binding pocket stabilizes the reaction intermediates and tames the propagation of carbocations through cation–π and other electrostatic interactions [54]. Moreover, TCs also assist intramolecular atom transfer and rearrangements including hydride or proton transfer and carbon shifts [10]. Eventually, the carbocation is quenched by deprotonation (E1-like) or nucleophilic attack (S_N_1-like) of water [45]. In contrast to sesquiterpenes (type I TCs), diterpenes can either be generated by type I TCs or type II TCs [11,55]. Type II TCs initiate carbocation formation by Brønsted acid catalysis to protonate a terminal isoprene double bond or an epoxide ring (Figure 5b) [56]. Thus, cyclization mediated by type II TCs leads to an inverted direction of charge propagation along the oligoisoprene chain in the cyclization cascade [53]. The resulting cyclized product retains the pyrophosphate moiety and can serve as the substrate for a second cyclization catalyzed by type I TCs to generate even more complicated diterpene backbones (Figure 5c).

Although the majority of terpene backbones results from the direct cyclization of FPP (**9**) and GGPP (**10**) catalyzed by either type I or mixed type II/I cyclases, there are cases known to divert from these canonical paths. The two homoterpene pathways, responsible for 2-methlyisoborenol [57] and sodorifen [58] biosynthesis for example, require the action of a methyltransferase preceding cyclization. Surprisingly, more and more terpene BGCs are characterized that do not harbor classical TCs, but instead use a variety of different enzyme classes for the cyclization reaction. These atypical terpene cyclization reactions have recently been extensively reviewed [53]. Many of these alternative cyclization routes have been shown to resemble classical type I and II cyclization mechanisms initiated by the formation of highly reactive short-lived carbocation intermediates (e.g., prenyltransferases [59–60], vanadium-dependent bromoperoxidases [61–62], or methyltransferases [63]).

In addition, enzymes typically classified as tailoring enzymes, such as flavin-dependent monooxygenases [64] and cytochrome P450s (CYPs) [65], were reported to be involved in noncanonical terpene cyclization. Furthermore, both oxidative [66] and reductive cyclizations [67] have been described. The reductive cyclization reaction is particularly noteworthy, as the reduction and cyclization step are catalyzed by two distinct enzymes [67]. These examples show that the classical division of terpene biosynthesis into a cyclization and decoration phase needs to be modified, as it is now known that enzyme families, traditionally regarded as tailoring enzymes, can potentially be involved in the cyclization reaction. The ever-increasing number of alternative terpene cyclization mechanisms suggests that nature has likely evolved additional, but so far undiscovered, modes of cyclization.

**Figure 6:**
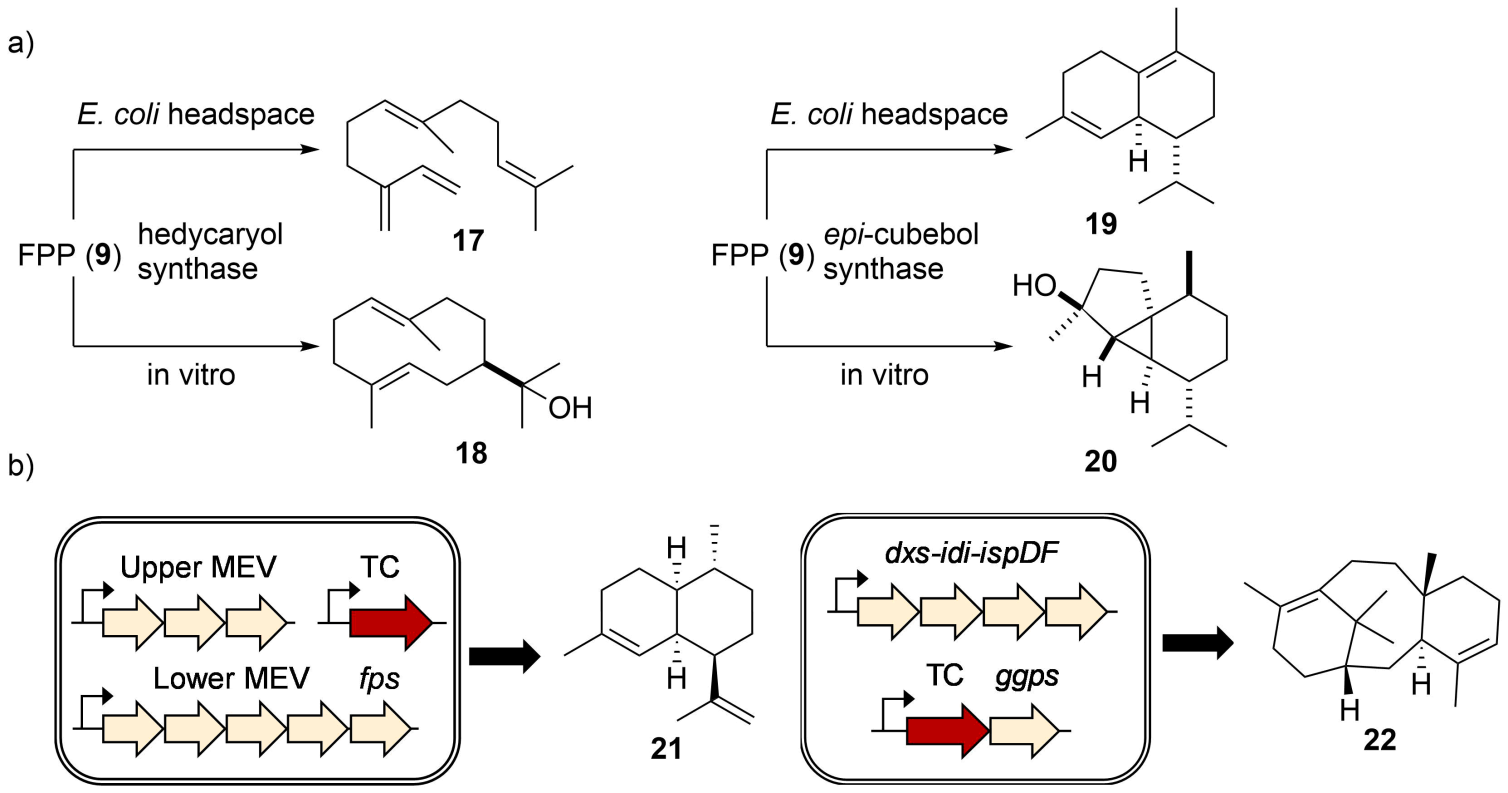
Functional TC characterization. a) Different terpenes were produced when hedycaryol (**18**) synthase and *epi*-cubebol (**20**) synthase were expressed in *E. coli* vs incubated with FPP (**9**) in vitro. b) Engineering the isoprenoid flux increases the titer of terpene production.

In vitro enzyme assays have been the method of choice for studying terpene cyclization (Supporting Information File 1, Table S1). The purified enzymes are particularly suitable for mechanistic investigations using strategically labeled substrates, as elegantly demonstrated in the studies on pentalenene synthase [68], geosmin synthase [69–71], and *epi*-isozizaene synthase [72]. Recently, all possible ^13^C-labeled FPP (**9**) [73] and GGPP (**10**) [74] precursors were synthesized and used to track the movement of carbon atoms and test mechanistic hypotheses. Using enantiotopically doubly ^13^C-^2^H-labeled substrates, it is possible to determine the stereochemistry of a cyclization product by locating the ^2^H atom and its relative position to other stereocenters [75]. In vitro terpene biosynthesis, however, might not always result in the production of the terpene skeleton produced natively or in the heterologous hosts (Figure 6a) [76]. In fact, it is occasionally observed that the same terpene cyclase generates different terpene skeletons dependent on the heterologous host in which it was expressed. While the exact reasons for the observed product variations are not fully understood, subtle differences in folding and reaction environment might be an explanation. In order to obtain larger quantities of terpenes, increasing the metabolic flux into isoprenoid pathways is often required. Overexpression of the bottleneck enzymes in the endogenous MEP pathway (*dxs*, *idi*, *ispD*, *ispF*, *fps/ggps*) or the entire exogenous MVA pathway in *E. coli* have been demonstrated to raise terpene titers dramatically [77] and have been applied for the production of several plant-derived terpene backbones, including amorpha-4,11-diene (**21**) [78–79] and taxadiene (**22**, Figure 6b) [80].

Despite efforts to characterize individual terpene cyclases and their modes of cyclization, no biosynthetic rules have so far been deduced that can be applied to a broad range of unrelated terpene cyclases.

### Phase 2) terpene scaffold functionalization

Tailoring enzymes are important elements that further increase the structural complexity of terpenoids by adding various functional groups. The size of bacterial terpene BGCs can vary significantly, which can be largely attributed to the presence or absence of tailoring genes. Some BGCs can be as large as modular thiotemplate BGCs (e.g., phenalinolactone (42 kb) [81] and platensimycin/platencin (47/41 kb) [82]) while most are less than 10 kb in size and harbor only one or two putative tailoring enzymes. Among them, oxidoreductases, especially CYPs, are the most abundant tailoring enzymes involved in the diversification process (Supporting Information File 1, Table S2).

CYPs are heme-dependent iron proteins that catalyze a wide range of reactions [83–84]. The reactions typically involve substrate radical generation by the activated iron species and subsequent hydroxylation. Terpenes are mainly composed of nonactivated hydrocarbons that are mostly chemically indistinguishable, while many P450s are known to selectively act on specific carbon atom(s). A continuous electron input is required to keep CYPs functional [83]. In general, soluble bacterial CYPs utilize class I redox systems, where the electron from NAD(P)H is delivered to CYPs through a ferredoxin reductase (FdR) and ferredoxin (FdX) [85]. Most bacteria have more than one FdR and FdX pair encoded in the genome [86]. It remains challenging to determine a priori which combination can reconstitute an active CYP, especially as the proximity of CYPs to these redox enzymes in the genome does not guarantee their partnership [87].

**Figure 7:**
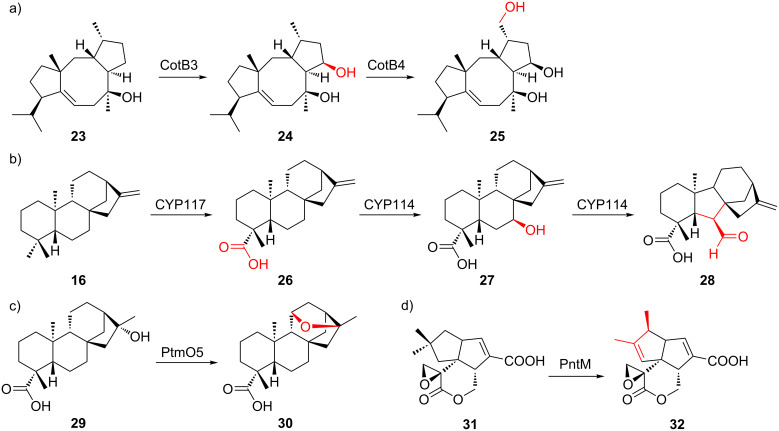
Selected examples of terpene modification by bacterial CYPs. a) Hydroxylation [89]. b) Carboxylation, hydroxylation, and ring contraction [41]. c) Ether formation [90]. d) Rearrangement [91].

Studies of CYPs in bacterial terpenoid biosynthesis lags behind those of cyclases, and only a handful of examples are found in literature (Supporting Information File 1, Table S2). CYPs mostly catalyze prototypical hydroxylations, sometimes followed by further oxidation to carbonyls or carboxylates. Other reactions, such as epoxidation, ether bond formation, and structural rearrangement have also been reported (Figure 7). CYP114 in gibberellin (**5**) biosynthesis, for example, catalyzes the unique oxidation/six-membered to five-membered ring contraction of *ent*-kaurenoic acid (**27** and **28**, respectively, Figure 7b) [41].

Characterization of CYPs is typically achieved by in vitro or in vivo studies. *E. coli* is the most popular host for obtaining proteins for in vitro studies, and proper selection of a redox system is usually the obstacle to reconstitute CYP activity. Substrates for in vivo studies are either added to the culture or supplied by coexpression of upstream enzymes in the heterologous hosts. If *E. coli* is selected as the host, coexpression of a redox system is required, as *E. coli* does not harbor any CYPs [88].

In comparison to TCs, tailoring genes, such as CYPs, can be annotated with high confidence, yet likewise no predictions about the function of the corresponding enzyme’s functions and stereo- or regiospecificity can be made. This lack of biosynthetic understanding is largely due to the relatively small number of characterized, bacteria-derived terpene-modifying enzymes. In addition, the fact that several CYPs have been shown to have relaxed substrate specificity, act on several intermediates, or catalyze multiple reactions, further complicates the in silico prediction.

### (Bio)synthetic production of complex terpenoids

**Figure 8:**
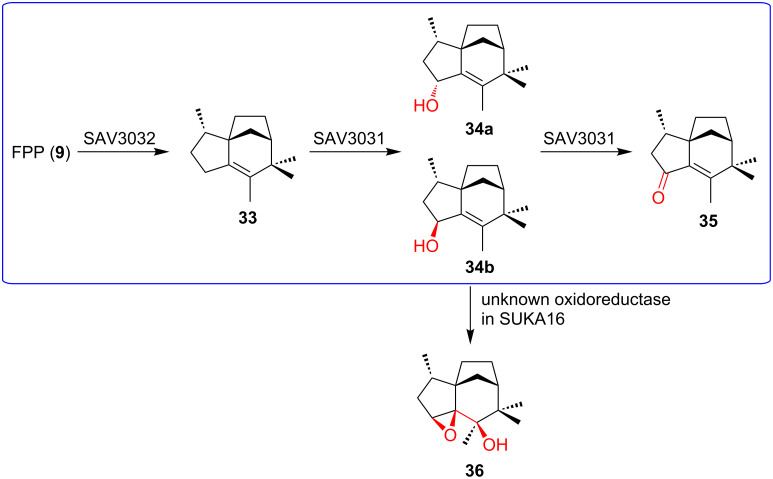
Off-target effects observed during heterologous expression of terpenoid BGCs. Unexpected oxidation of **34b** by an oxidoreductase in the heterologous host *S. avermitilis* SUKA16. The native pathway is highlighted in blue.

Heterologous expression is the most widely used method to study complex terpenoid biosyntheses. Since many bacterial terpenoid BGCs are actinomycete-derived, terpene BGCs are often expressed in model *Streptomyces* hosts, such as *Streptomyces albus*, *Streptomyces avermitilis*, *Streptomyces coelicolor*, and *Streptomyces lividans*, under the control of exogenous promoters [92–94]. To minimize the cellular resource competition and facilitate cleaner analysis, many of these hosts have been engineered to remove native secondary-metabolite BGCs and for optimized terpene precursor supply [1,95–97]. Like *Streptomyces* hosts, many *E. coli* hosts have been engineered to increase precursor supply, provide redox partners for CYP enzymes, and overproduce oligoprenyl diphosphate. As is the case for other natural product classes, off-target effects of heterologous host enzymes have been reported to alter terpenoid structures significantly (Figure 8) [98].

Despite difficulties in engineering terpene biosynthesis, total synthesis of complex terpenoids does not appear to be a viable alternative. Traditionally, complex terpenes are synthesized from small and cheap chiral terpenes as starting material in (linear) multistep syntheses [99]. This way, pre-existing oxidized functionalities need to be maintained and propagated with complex protecting group strategies en route to the desired target [100]. Most importantly, however, these total syntheses can easily require 50 or more steps, with poor overall yields, and are hence far away from nature’s efficiency and pathway-encoded diversity [47,101]. The insights gained into the biosynthesis of complex terpenes have resulted in alternative approaches towards complex terpenoids – biomimetic syntheses. Cyclization reactions have been successfully mimicked using a wide variety of conditions. In analogy to nature’s biosynthetic strategies outlined above, cationic, Diels–Alder, oxidative, and radical cyclization strategies have been successfully applied in total syntheses of terpenoids [53]. In addition, chemists are currently creatively exploring means to mimic classical terpene cyclases and their chaperone-like properties [102–104]. Unlike the generation of hydrocarbon backbones in nature, even the biomimetic synthetic construction of terpene scaffolds is usually a multistep process [17]. The second phase of a biomimetic synthesis, in analogy to terpene biosynthesis, involves the challenging introduction of functional groups (mostly hydroxy groups) into nonactivated C–H bonds [46,105–112]. The absence of directing functional groups, however, renders these regioselective oxidations of nonactivated carbon atoms highly challenging, as the chemical functionalization of nonactivated carbon atoms is one of the remaining challenges in organic synthesis [100,112–113]. Despite the progress made in total synthesis of terpenoids and the introduction of the two-phase mimicry of terpene biosynthesis during biomimetic syntheses, it has become obvious that total synthesis is currently not an efficient alternative to either the biotechnological production of terpenoids or the biosynthetic expansion of terpene chemical space [100,113].

### Engineering terpene biosynthetic features to produce novel, non-natural terpenoids

#### Terpene cyclases

**Figure 9:**
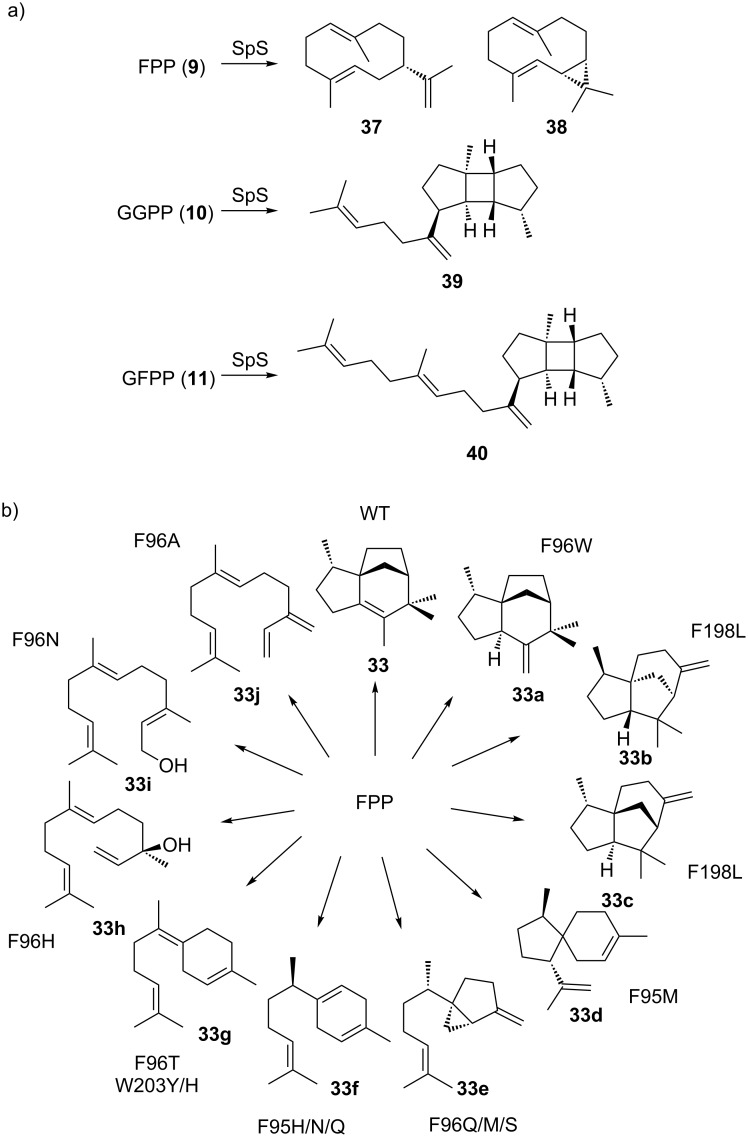
TC promiscuity and engineering. a) Spata-13,17-diene (**39**) synthase (SpS) can take C_15_ and C_25_ oligoprenyl diphosphate as substrates in addition to its C_20_ natural substrate. b) Selected examples of *epi*-isozizaene (**33**) synthase mutants that produce different sesquiterpene skeletons.

Most bacterial TCs produce one major product, while few of them are known to produce multiple products [1,50,76,114–117]. Among them, 10-*epi*-cubebol synthase from *Sorangium cellulosum* Soce56 is the most prolific bacterial TC known. It produces at least 25 different sesquiterpene skeletons in addition to 10-*epi*-cubebol [116]. In some cases, bacterial TCs are able to accept oligoprenyl diphosphates with different lengths. Spata-13,17-diene (**39**) synthase is an extreme example that can convert FPP (**9**), GGPP (**10**), and geranylfarnesyl diphosphate (GFPP, **11**) into sesquiterpenes, diterpenes, and sesterterpenes, respectively (**37**–**40**), though only diterpenes were observed in the culture headspace of the wild type producer (Figure 9a) [118]. These examples suggest a remarkable degree of plasticity of TC active sites in order to direct the intermediate to different trajectories and accommodate substrates with variable lengths. Indeed, besides the influence on kinetic properties by changing the conserved motifs, many bacterial TCs are able to produce novel skeletons through mutations of other active-site residues. This could result in either the arrest of catalytic intermediates or the creation of new trajectories to quench the cationic species. For instance, remodeling the hydrophobic pocket of the active site by single-point mutation, *epi*-isozizaene (**33**) synthase was engineered to produce various linear, monocyclic, bicyclic, and tricyclic terpene skeletons (Figure 9b) [119–121]. Another prominent example is cyclooctat-9-en-7-ol (**23**, Figure 7a) synthase, for which the active-site residues responsible for cationic intermediate stabilization were identified through analysis of the crystal structure [122] and structural modeling [123]. Mutations of the identified residues were shown to alter product profiles and yielded several new terpene skeletons. With the growing mechanistic and structural knowledge of bacterial TCs, and the fact that many TCs naturally produce multiple products, there is great potential for the discovery of novel skeletons or enhancing the production of desired backbones among other side products, simply by rationally mutating the active-site residues.

The majority of bacterial type II diterpene TCs produce bicyclic labdane, halimadane, or clerodane skeletons with different stereochemistry, levels of unsaturation, and hydroxylation patterns [124], which undergo further conversion with their cognate type I diterpene TCs. It was therefore hypothesized that promiscuous type I TCs might be able to process different type II products to generate non-natural structural diversity. Indeed, pairing of type II TCs with type I TCs generated 13, 41, and 49 novel terpene skeletons, respectively, in three separate studies [125–127]. Though mostly plant TCs were employed, the few bacterial cyclases tested were also shown to be productive, suggesting this promiscuity is not limited to plant TCs. Type II/I diterpene TC tandems, however, are less prevalent in bacteria [11,55]. With the ever-increasing number of bacterial genome sequences available and the development of advanced bioinformatics tools, more type II/I diterpene TC tandems are likely to be discovered. By applying the same combinatorial concept, it is expected that the diterpene chemical space can be significantly expanded.

#### Cytochrome P450s

**Figure 10:**
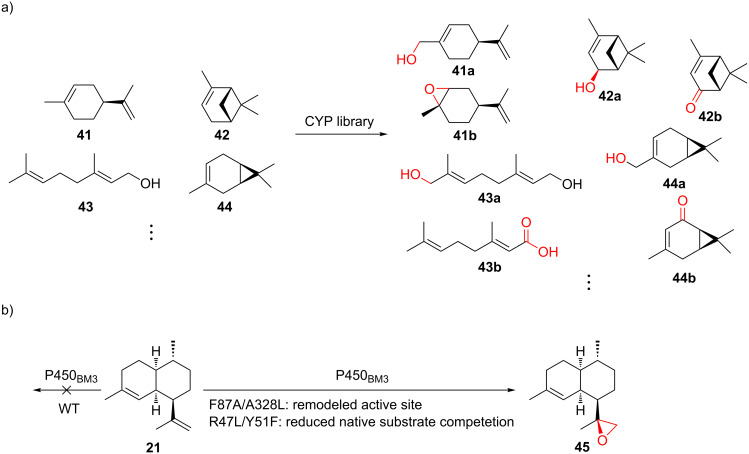
Substrate promiscuity and engineering of CYPs. a) Selected examples from using a CYP library to oxidize various monoterpenes. b) Rational engineering of P450_BM3_ for epoxidation of amorphadiene (**21**). F87A/A328L and R47L/Y51F: mutations.

CYPs have the ability to install functional groups at nonactivated C–H bonds with exceptional selectivity. In contrast to the common perception of enzymes being highly substrate-specific, many CYPs are able to modify non-natural substrates but retain their high stereo- and regiochemical selectivity observed from the transformation of their natural substrate. For the exploration of substrate promiscuity of plant-derived *ent*-kaurene oxidases, twenty combinations of type II/I diterpene TCs were coexpressed with one of the two *ent*-kaurene oxidases (CYPs) and their native CPRs from different plant sources, which led to the production of 12 novel oxidized labdane-related diterpenes [128]. Recently, commercially available monoterpenes were incubated with cell-free extracts from *E. coli* expressing well-studied bacterial CYPs, resulting in 27 previously unreported terpenoids (selected examples are shown in Figure 10a) [129]. While only a limited number of bacterial CYPs for terpene modification has been characterized, many of them were reported to exhibit substrate promiscuity (Supporting Information File 1, Table S2). These promiscuous CYPs do not necessarily co-localize with TC-encoding genes in the genomes, suggesting their potential to diversify terpenes out of their biosynthetic contexts.

Directed evolution of CYPs and other heme-containing proteins has a long history [130] and could potentially be applied to terpenoid diversification. Rational design, on the other hand, relies on crystal structures, homology models, or mechanistic information to select the residues to be mutated. For example, P450_BM3_ (CYP102A1) was rationally evolved to catalyze the epoxidation of amorphadiene (**21**) by expanding the active site, minimizing competing reactions, and facilitating substrate access (Figure 10b) [131]. Similarly, by mutating two residues of P450_BM3_ that control the shape of the substrate binding cavity, the enzyme was engineered to accept different linear, monocyclic, and bicyclic terpenes [132]. The alteration of the native sequences uses either method or the combination of the two, serving as an indispensable tool to introduce another dimension for terpenoid diversification.

### Expanding terpene chemical space through pathway engineering

**Figure 11:**
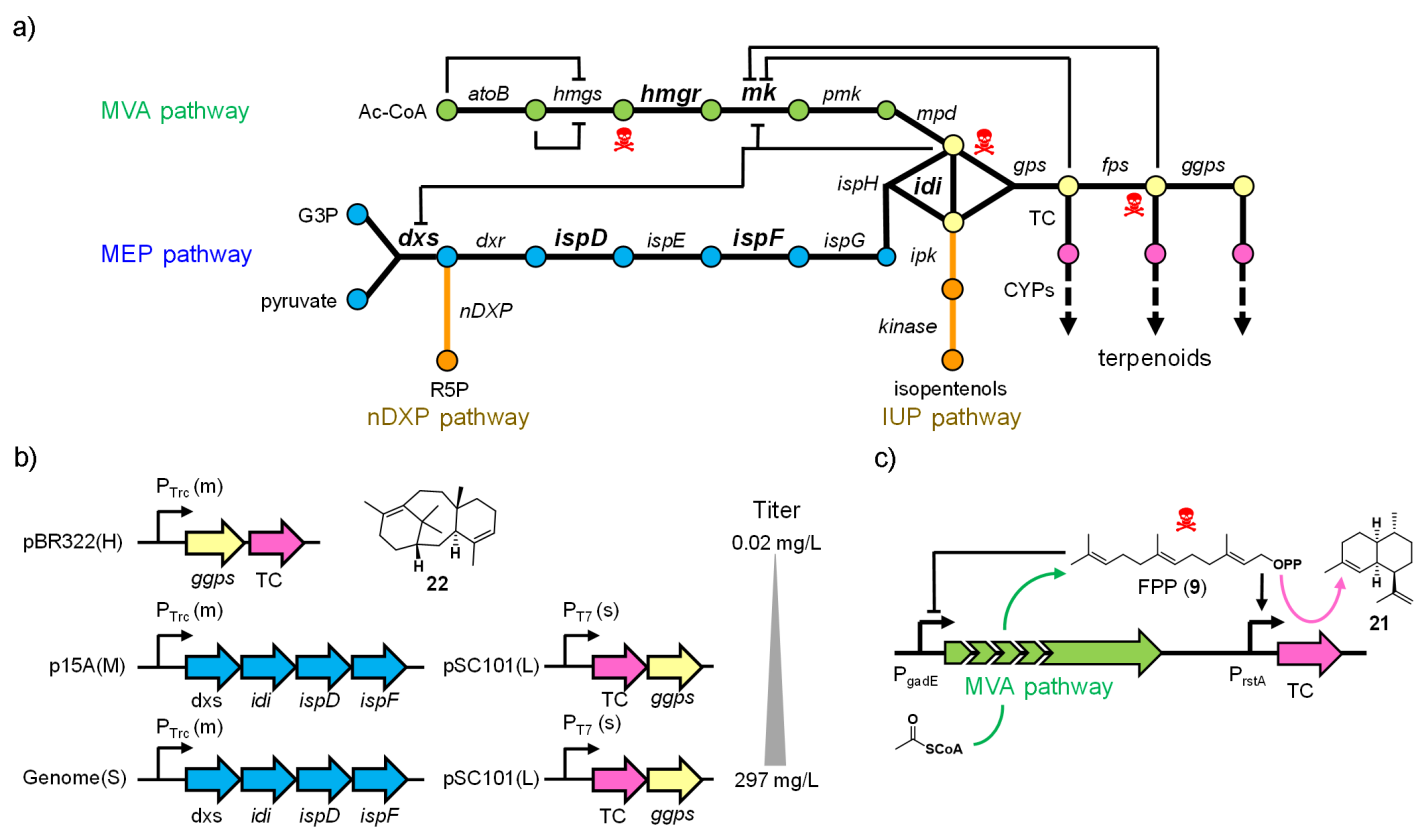
Engineering of terpenoid pathways. a) Metabolic network of terpenoid biosynthesis. Toxic intermediates are labeled with skull signs and known enzyme inhibition by intermediates is indicated. Bottleneck enzymes that have been subjected to optimization/engineering are highlighted in bold. Two novel pathways, nDXP and IUP, provide alternative entry points to terpenoid biosynthesis. b) Balancing gene expression for taxadiene (**22**) production in *E. coli*. The copy number of the constructs are labeled with upper case letters: H: high, M: medium, L: low, and S: single copy. The relative strength of the promoter is labeled with lower case letters: s: strong and m: medium. This tuning led to 15,000-fold increase in taxatidene titer. c) Two FPP (**9**)-responsive promoters were implemented to dynamically control the expression of MVA pathway genes and the terpene cyclase gene in order to prevent the accumulation of toxic FPP (**9**).

Engineering of terpenoid biosynthetic pathways has focused on enhancing the metabolic flux to supply isoprenoid precursors (Figure 11a) [77,133]. Common strategies include expressing an entire exogenous isoprenoid pathway (e.g., the MVA pathway in *E. coli*) and overexpressing bottleneck enzymes (e.g., *dxs*, *idi*, *ispD*, *ispF* in the MEP pathway; *mk*, *hmgr* in the MVA pathway) [78–80,134–136]. Attempts are also being made to search for and engineer more active pathway enzyme variants [136–138]. As the balanced pathway expression is often the key to high terpenoid titers, these pathway genes have been grouped into “modules”, using a multivariate-modular approach [80], in vitro enzyme activity assays [139], proteomics-based principal component analysis [140], qPCR/proteomics [141], and iterative grid search [142] to troubleshoot and guide the tuning of the promoter strengths and copy numbers of each module/enzyme (Figure 11b).

Toxicity of biosynthetic intermediates, endogenous regulation, and stability of genetic constructs are the main concern during these engineering efforts. IPP (**6**), FPP (**9**), and HMG-CoA (3-hydroxy-3-methylglutaryl-CoA) are known to be toxic [78,143], and FPP-dependent stress-responsive promoters have recently been identified through microarray experiments to dynamically regulate pathways and minimize the accumulation of FPP (Figure 11c) [143]. Alternatively, in vitro pathway reconstitution led to the successful production of several monoterpenes, circumnavigating toxicity to the heterologous hosts [144]. Several intermediates are also known to inhibit enzyme activities in the pathway. Novel pathways, such as the novel 1-deoxy-ᴅ-xylulose 5-phosphate pathway (nDXP) [145] and the isopentenol utilization pathway (IUP) [146], that bypass (part of) the natural MVA or MEP pathway were designed to circumvent this complex endogenous interaction and regulation (Figure 11a). Lastly, integration of pathways into the *E. coli* genome and the subsequent tuning via recombineering or CRISPR/Cas9 tools has been pursued to solve the instability of plasmid-based systems [147–148].

The comparably small size of terpenoid BGCs, the promiscuity of the enzymes involved, and the fact that the core is usually built up in one step and then modified by tailoring reactions make terpenoid biosynthesis particularly amenable for generating non-natural variants through engineering. It is easy to imagine that the combinatorial pairing of promiscuous TCs and CYPs harbors great potential to achieve this goal, and the integration of machine learning and retrobiosynthetic algorithms could facilitate the design of constructs for specific terpenoid variants [149]. While it is now relatively straightforward to direct the flux to produce terpene skeletons, less is known about how to effectively support function of CYPs beyond natural/surrogate redox partners or fusing CYPs with redox partners [80,136,141,150–151]. One common observation is that CYP activity is not able to match the high flux of isoprenoids, leaving a significant portion of terpenes unmodified. Solutions to this problem, such as in silico redox partner prediction, remain to be explored [152].

One major bottleneck in these studies is the lack of high-throughput screening tools for terpenoid production and characterization. Most terpenoids do not have any chromogenic groups and their detection still relies on liquid chromatography–mass spectrometry setups, limiting the number of screens that can be done. Though genetic construction, culturing bacteria, terpenoid extraction, and chromatographic analysis can be carried out (semi-)automatedly, terpenoid structure elucidation constitutes another major bottleneck of these endeavors. At this end, the development of powerful chemoinformatic platforms is likely to assist in detecting putative terpenoids.

In the future, synthetic biology will likely allow the expansion of terpene chemical space by efficiently producing novel terpenes in genome-optimized host strains and the generation of non-natural terpenes through the fine control of terpene cyclases and tailoring enzymes. The reduced cost of DNA synthesis [153] and advanced cloning techniques [154], such as Gibson Assembly [155] and Golden Gate cloning [156], has made pathway construction more economically efficient, and the genetic parts can also be retrieved, recombined, and reused easily for different applications. Insights into terpene biosynthesis, along with advancements in synthetic biology, will pave the way towards the sustainable and selective biotechnological production of high-value terpenes, such as taxol (**1**). One recent study describes the successful reconstitution of THC (**3**) biosynthesis in yeast by upregulating MVA pathway genes, mutating *erg20* (*fps*) in favor of GPP (**8**) over FPP (**9**) production, using genes from multiple different species to supply hexonyl-CoA, and identifying key coupling enzyme through bioinformatic analysis [157]. THC variants could also be obtained via feeding the engineered strain with substrate analogs. This integration of multiple strategies highlights the great potential of synthetic biology for the production of valuable complex terpenoids [157].

## Conclusion

Despite the ubiquitous distribution of terpene biosynthetic pathways in bacteria, only a few terpenes of bacterial origin have been characterized, and their biosynthesis is for the most part poorly understood. The lack of bio-/chemoinformatics platforms to predict terpene core structures coupled with their physicochemical properties have rendered the targeted isolation of complex terpenoids from their native producers highly challenging. Since terpene biosynthetic pathways are comparably small, heterologous expression of entire pathways in suitable host strains is the method of choice to retrieve the biosynthetic treasures hidden in bacterial genomes. Moreover, the biosynthetic logic of terpene biosynthetic pathways with a single enzyme for hydrocarbon backbone cyclization and multiple tailoring enzymes is ideally suited for synthetic biology approaches to engineer new pathways. These new pathways would enable the combinatorial expansion of terpene chemical space and the creation of non-natural biosynthetic pathways for the production of high-value terpenoids, especially pharmaceutical agents. The development of highly manipulable host strains with multiple orthogonally inducible promoters allows for the tight control, timing, and analysis of these combinatorial endeavors. In combination with directed mutagenesis to relax, alter, and expand cyclization, tailoring patterns, and substrate scope, synthetic biology is ideally suited to generate non-natural terpenes with promising drug-like properties and biological activities. Results obtained from these studies will also assist in refining our understanding of bacterial terpenoid biosynthesis.

## Supporting Information

File 1 Additional figures and tables.
