# Supplementary material for: Bacterial terpene biosynthesis: challenges and opportunities for pathway engineering
Source: Beilstein J Org Chem. 2019 Nov 29;15:2889–906. doi: 10.3762/bjoc.15.283 (PMC6902898; doi:10.3762/bjoc.15.283)
Supplement: File 1 — Additional figures and tables. [file Beilstein_J_Org_Chem-15-2889-s001.pdf]

BEILSTEIN JOURNAL OF ORGANIC CHEMISTRY

Supporting Information

for

Bacterial terpene biosynthesis: challenges and opportunities for pathway engineering

Eric J. N. Helfrich, Geng-Min Lin, Christopher A. Voigt and Jon Clardy

Beilstein J. Org. Chem. **2019,** *15,* 2889–2906. doi:10.3762/bjoc.15.283

Additional figures and tables

License and Terms: This is a supporting information file under the terms of the Creative Commons Attribution License (http://creativecommons.org/licenses/by/4.0). Please note that the reuse, redistribution and reproduction in particular requires that the authors and source are credited.
The license is subject to the *Beilstein Journal of Organic Chemistry* terms and conditions: (https://www.beilstein-journals.org/bjoc)

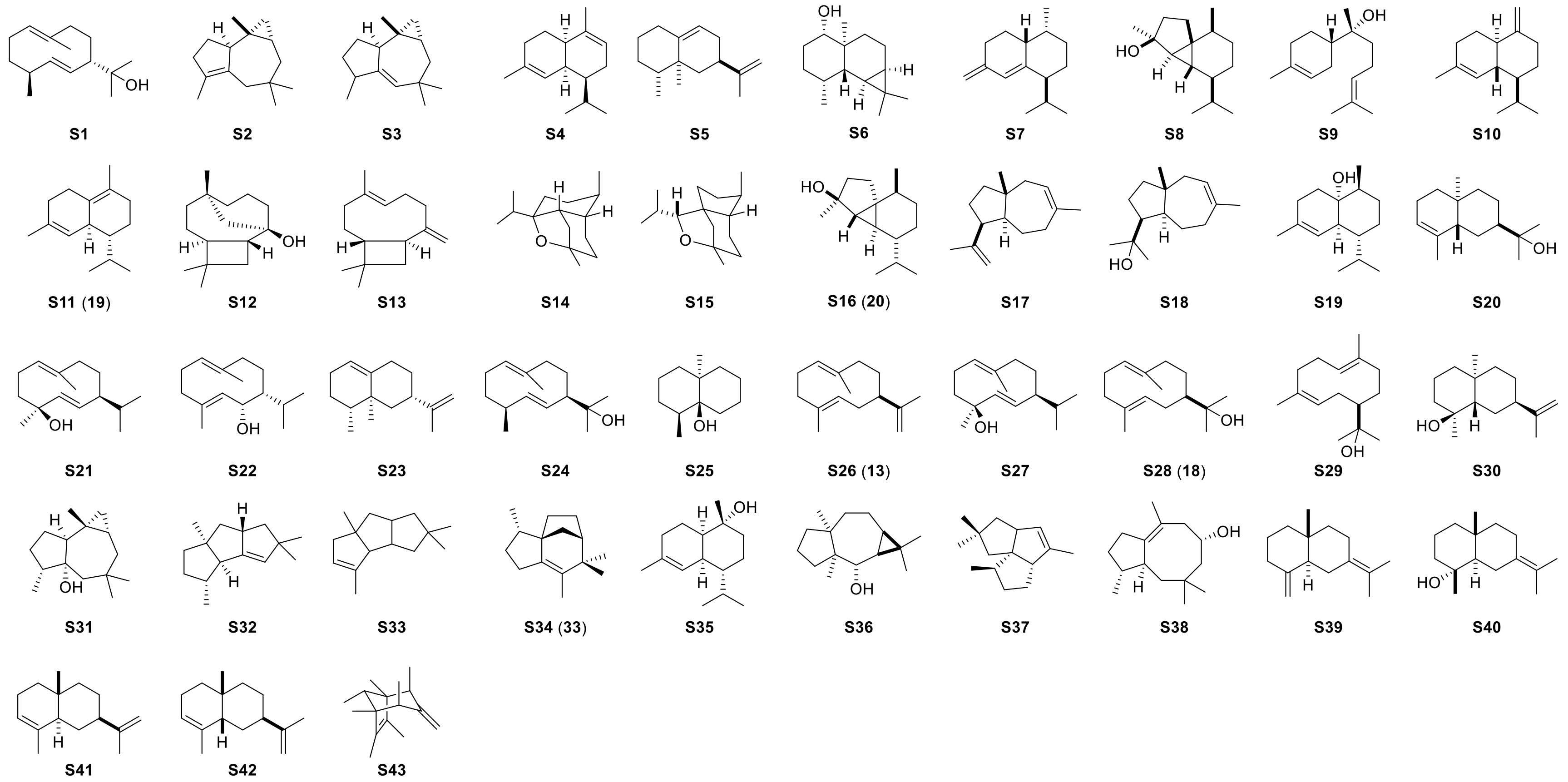

Figure S1: Characterized bacterial sesquiterpenes.

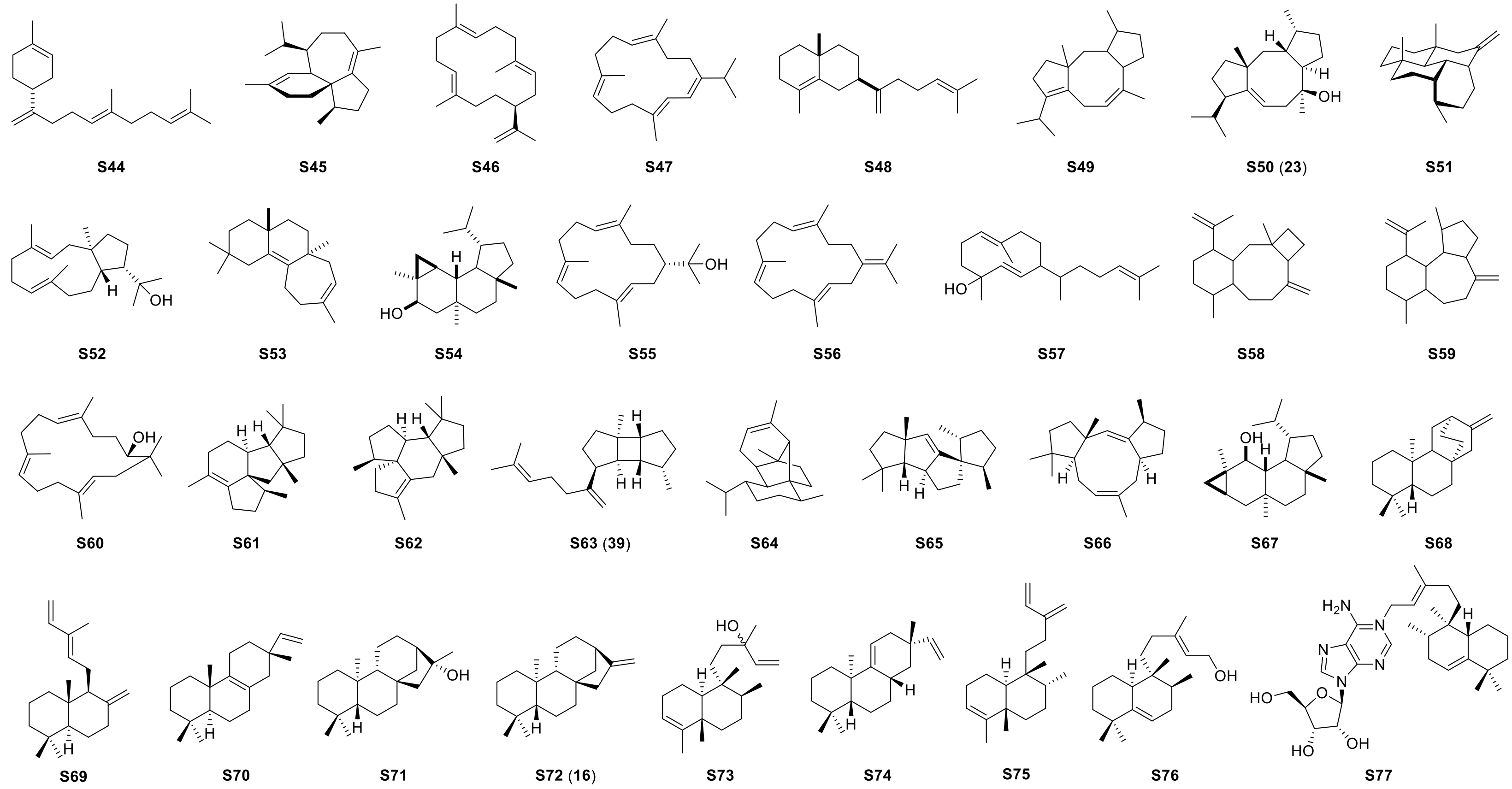

Figure S2: Characterized bacterial diterpenes.

Table S1: Characterized bacterial terpene cyclases.

Terpene[a]	Uniprot #	Name	Producing Strain	Host[b]	Study[c]	Isoprenoid Flux	Ref
**Sesquiterpene**							
(+)-allohedycaryol (**S1**)	B2HGU3	mmar_3220	*Mycobacterium marinum*	SUKA22	B	*fps*	1
african-2-ene (**S2**)* / african-1-ene (**S3**)*	D5SKM9	sclav_p0985	*Streptomyces clavuligerus*	SUKA22	B	*fps*	1
(-)-α-amorphene (**S4**)	D9XDR8		*Streptomyces viridochromogenes*	BL21(DE3)	A/B	endogenous	2-4
aristolochene (**S5**)*	A0A117E6J5		*Streptomyces acidiscabies*	WT			5
avermitilol (**S6**)	Q82RR7	sav76	*Streptomyces avermitilis*	BL21(DE3)	A	N/A	5
				SUKA17	B	*fps*	5
bicyclosesquiphellandrene (**S7**)*	A9GK58	sce6369	*Sorangium cellulosum*	SUKA22	B	*fps*	1
10-*epi*-cubebol (**S8**)*[,e]				C43(DE3)	B	MEV pathway	6
(-)-*epi*-α-bisabolol (**S9**)	G5EKN0	SC2	*Streptomyces citricolor*	BL21(DE3)	A	N/A	7
				TK21	B	endogenous	7
(-)-γ-cadinene (**S10**)	C7PLV2		*Chitinophaga pinensis*	BL21(DE3)	A/B	endogenous	2, 3
(-)-δ-cadinene (**S11**, **19**)	B5GS26	sscg_02150/ sclav_p0328	*Streptomyces clavuligerus*	BL21(DE3)	A	N/A	8
				JM109(DE3)	B	MEV pathway	8
				SUKA22	B	*fps*	1
(+)-caryolan-1-ol (**S12**)	B1W019	gcoA	*Streptomyces griseus*	BL21(DE3)	A	N/A	9
				TK21	B	endogenous	9
				SUKA22	B	*fps*	1
(-)-(*E*)-β-caryophyllene (**S13**)	K0K750		*Saccharothrix espanaensis*	BL21(DE3)	A/B	endogenous	3, 10
corvol ether A (**S14**)* / corvol ether B (**S15**)*	E4N7E5		*Kitasatospora setae*	BL21(DE3)	A	N/A	4, 11, 12
epi-cubebol (**S16**, **20**)*	D2B747		*Streptosporangium roseum*	BL21(DE3)	A/B	endogenous	3, 4, 10, 12
(+)-dauca-8,11-diene (**S17**)* / (+)-isodauc-8-en-11-ol (**S18**)*	F2R8C1	sven_0552	*Streptomyces venezuelae*	SUKA22	B	*fps*	1
				BL21(DE3)	A	N/A	12, 13
(+)-epicubenol (**S19**)	B1W477	gecA	*Streptomyces griseus*	BL21(DE3)	A	N/A	14
				TK21	B	endogenous	14
				SUKA22	B	*fps*	1
(+)-eremophilene (**S20**)	A9FZ87	geoA/ sce8852	*Sorangium cellulosum*	BL21(DE3)	A	N/A	15

				C43(DE3)	B	endogenous	15
				SUKA22	B	*fps*	1
7-*epi*-α-eudesmol (**S21**)*	D9XD61		*Streptomyces viridochromogenes*	BL21(DE3)	A/B	endogenous	2, 3
(-)-germacradien-4-ol (**S22**)	G5EKM9	SC1	*Streptomyces citricolor*	BL21(DE3)	A	N/A	7
				TK21	B	endogenous	7
	A0A097ZQD8	slt18_1246	*Streptomyces lactacystinaeus*	SUKA22	B	*fps*	1
germacradien-6-ol (**S23**)	E8W6C7		*Streptomyces pratensis*	BL21(DE3)	A	N/A	4, 16
1(10),5-germacradien-11-ol (**S24**)/ geosmin (**S25**)	Q9X839	sco6073/ ScGS	*Streptomyces coelicolor*	pLysS	A	N/A	17-21
	Q82L49	sav2163/ geoA	*Streptomyces avermitilis*	BL21(DE3)	A	N/A	22
	B2IUI7	NP2	*Nostoc punctiforme*	JM109	B	endogenous	23
				pLysS	A	N/A	24
	B0FLN6	spterp13	*Streptomyces peucetius*	BL21(DE3)	A	N/A	25
1(10),5-germacradien-11-ol (**S24**)*[,d]	A9F845	sce1440	*Sorangium cellulosum*	C43(DE3)	B	endogenous	6
germacrene A (**S26**, **13**)*	Q8YN85	NS1/ alr4685	*Nostoc sp.* PCC 7120	JM109	B	endogenous	23
				BL21(DE3)	A	N/A	23
				SUKA22	B	*fps*	1
	Q3MBN2	ava_1982	*Anabaena variabilis*	SUKA22	B	*fps*	1
(4*S*,7*S*)-germacrene D-4-ol (**S27**)	A0A127Q4J7		*Collimonas pratensis*	BL21(DE3)	A	N/A	4
hedycaryol (**S28**, **18**)*	A0A2N3Y098		*Saccharopolyspora spinosa*	BL21(DE3)	A/B		10
(2Z,6E)-hedycaryol (**S29**)*	E4MYY0	HcS	*Kitasatospora setae*	BL21(DE3)	B	endogenous	26
(+)-intermedeol (**S30**)	B5GTQ6	sclav_p0635	*Streptomyces clavuligerus*	SUKA22	B	*fps*	1
				BL21(DE3)	A	N/A	13
isoafricanol (**S31**)	G2P5T1		*Streptomyces violaceusniger*	WT			27
	A0A291SJC7		*Streptomyces malaysiensis*	BL21(DE3)	A	N/A	28
isohirsut-1-ene (cucumene, **S32**)	B5GLM7		*Streptomyces clavuligerus*	SUKA22	B	*fggps*	1, 29
				BL21(DE3)	A	N/A	30
isohirsut-4-ene (**S33**)*	A0A097ZQA4	slt18_1880	*Streptomyces lactacystinaeus*	SUKA22	B	*ggps*	1, 29
(+)-*epi*-isozizaene (**S34**, **33**)	Q9K499	sco5222	*Streptomyces coelicolor*	BL21(DE3)	A	N/A	31-34
	Q82IV1	sav3032	*Streptomyces avermitilis*	BL21(DE3)	A	N/A	35
(+)-T-muurolol (**S35**)	B5GW45	sscg_03688/ sclav_p0068	*Streptomyces clavuligerus*	BL21(DE3)	A	N/A	8
				JM109(DE3)	B	MEV pathway	8
				SUKA22	B	*fps*	1
	A7NH01		*Roseiflexus castenholzii*	BL21(DE3)	A/B	endogenous	2-4, 36

	A5UZ14	roseRS_3509	*Roseiflexus sp.* RS-1	SUKA22	B	*fps*	8
neomeranol B (**S36**)*	C9Z4F7		*Streptomyces scabiei*	BL21(DE3)	A	N/A	13
pentalenene (**S37**)	Q55012	PenA	*Streptomyces exfoliatus*	BL21(DE3)	A	N/A	37, 38
				XD	B	endogenous	39
	Q82IY4	sav2998/ PtlA	*S. avermitilis*	BL21(DE3)	A	N/A	40
pristinol (**S38**)	B5H7H3		*Streptomyces pristinaespiralis*	BL21(DE3)	A	N/A	41
selina-4(15),7(11)-diene (**S39**)*	L8ERG6		*Streptomyces rimosus*	WT			42
	B5HDJ6		*Streptomyces pristinaespiralis*	BL21(DE3)	A/B	endogenous	2
selina-7(11)-ene-4-ol (**S40**)*[,d]	A0A097ZQE4	sspSK_3051	*Streptomyces* sp. SK 1894	SUKA22	B	*fps*	1
α-selinene (**S41**)*	A9B3Q4	haur_2988	*Herpetosiphon aurantiacus*	SUKA22	B	*fps*	1
5-*epi*-α-selinene (**S42**)*	B2J4A4	NP1/ npun_R3832	*Nostoc punctiforme*	JM109	B	endogenous	23
				BL21(DE3)	A	N/A	23
				SUKA22	B	*fps*	1
sodorifen (**S43**)[e]	A0A318P116/ A0A318P5Q0	sod_c20750/ sod_c20760	*Serratia plymuthica* 4Rx13	BL21(DE3)	A/B	N/A	43, 44
**Type I Diterpene**							
axinyssene (**S44**)*[#]	A0A084SQV5	CysTC2	*Archangium violaceum*	BW25113	B	MEV pathway	45
bonnadiene (**S45**)	A0A1G9UQQ0	BdS	*Allokutzneria albata*	BL21(DE3)	A	N/A	46
(*S*)-cembrene A (**S46**)	A0A1G9S4L4	CAS	*Allokutzneria albata*	BL21(DE3)	A	N/A	47
cembrene C (**S47**)	Q1AYR1	rxyl_0493	*Rubrobacter xylanophilus*	SUKA22	B	*ggps*	1
clavulatriene A (**S48**)*[,d]	B5H135	sclav_p1169	*Streptomyces clavuligerus*	SUKA22	B	*ggps*	1, 29
cyclooctat-7(8),10(14)-diene (**S49**)*	A0A097ZQD0	slt18_1078	*Streptomyces lactacystinaeus*	SUKA22	B	*ggps*	1, 29
cyclooctat-9-en-7-ol (**S50**, **23**)	C9K1X5	CotB2	*Streptomyces melanosporofaciens*	BL21(DE3)	A	N/A	48, 49
				S. albus	B	*ggps*	48
				BL21(DE3)	A/B	*dxs/dxr/idi/ggps*	49-51
				Rosetta2	A	N/A	52
hydropyrene (**S51**)	D5SK09	sclav_p0765/ HpS	*Streptomyces clavuligerus*	SUKA22	B	*ggps*	1, 29
				BL21(DE3)	A	N/A	53
18-hydroxydolabella-3,7-diene (**S52**)	C7PA56	HdS	*Chitinophaga pinensis*	BL21(DE3)	A	N/A	54
				NB	B	endogenous	54
lydicene (**S53**)*[#]	A0A0D4DXU3	StlTC	*Streptomyces lydicus*	BW25113	B	MEV pathway	45
neoverrucosan-5β-ol (**S54**)*[#]	H6KZW8	SapTC1	*Saprospira grandis*	BW25113	B	MEV pathway	45

(*R*)-nephthenol (**S55**) / cembrene C isomer (**S56**)	M1V9Q0	DtcycA	*Streptomyces* sp. SANK 60404	BL21(DE3)	A	N/A	55
obscuronatin (**S57**)*	A9B3Q3	haur_2987	*Herpetosiphon aurantiacus*	SUKA22	B	*ggps*	1
odyverdiene A (**S58**)* / odyverdiene B (**S59**)*	A0A097ZQD1	nd90_0354	*Streptomyces sp.* ND90	SUKA22	B	*ggps*	1, 29
**S60**[d]	M1VDX3	DtcycB	*Streptomyces sp.* SANK 60404	BL21(DE3)	A	N/A	55
phomopsene (**S61**)/ allokutznerene (**S62**)	A0A1G9VRW7	PmS	*Allokutzneria albata*	BL21(DE3)	A	N/A	46
spata-13,17-diene (**S63**, **39**)	UPI000BAF81BA	SpS	*Streptomyces xinghaiensis*	BL21(DE3)	A	N/A	36, 56
spiroalbatene (**S64**)	A0A1H0BVV7	SaS	*Allokutzneria albata*	BL21(DE3)	A	N/A	36, 46
(-)-spiroviolene (**S65**)	P0DPK6	SvS	*Streptomyces violens*	BL21(DE3)	A	N/A	57
tsukubadiene (**S66**)	I2N045	stsu_20912	*Streptomyces tsukubensis*	SUKA22	B	*ggps*	1, 29
				BL21(DE3)	A	N/A	36, 57
verrucosane-2β-ol (**S67**)*[#]	A9WGY1	ChlTC2	*Chloroflexus aurantiacus*	BW25113	B	MEV pathway	45
	A0A178MLU0	ChlTC5	*Chloroflexus islandicus*	BW25113	B	MEV pathway	45
**TypeII/I Diterpene**[f]							
ent-atiserene (**S68**)	DBL2U6/ DBL2U4	PtmT2/ PtmT1	*Streptomyces platensis*	BL21(DE3)	A/B	*dxs/dxr/idi/ggps*	58, 59
biformene (**S69**)	A0A0H5BB10/ A0A0H5BN57	CldB/ CldD	*Streptomyces cyslabdanicus*	JM109(DE3)	A	NA	60, 61
				SUKA22	B	*ggps*	
isopimara-8,15-diene (**S70**)	A8M709/ A8M708	sare_1288/ sare_1287	*Salinispora arenicola*	C41(DE3)	B	*ggps*	62
(16*R*)-*ent*-kauran-16-ol (**S71**)	DBL2U6/ DBL2U9	PtmT2/ PtmT3	*Streptomyces platensis*	BL21(DE3)	A/B	*dxs/dxr/idi/ggps*	58, 59
ent-kaur-16-ene (**S72**, **16**)	Q45221/ Q45222	blr2149/ bl42150	*Bradyrhizobium japonicum*	C41(DE3)	B	endogenous	63
	Q989L7/ Q989L6		*Mesorhizobium japonicum*	C41(DE3)	B	*dxs/dxr/idi/ggps*	64
	P55538/ P55537		*Sinorhizobium fredii*	C41(DE3)	B	*dxs/dxr/idi/ggps*	64
	Q8KLD5/ Q8KLD4		*Rhizobium etli*	C41(DE3)	B	*dxs/dxr/idi/ggps*	64
(+)-kolavelool (**S73**)	A9AWD5/ A9AWD6	haur_2145/ haur_2146	*Herpetosiphon aurantiacus*	BL21(DE3)	A	N/A	65

ent-pimara-9(11),15-diene (**S74**)	Q5KSN5/<br>Q5KSN4		*Streptomyces sp.* KO-3988	M15/pREP4	A	N/A	66, 67
terpentetriene (**S75**)	Q9AJE4/<br>Q9AJE3		*Kitasatospora griseola*	M15/pREP4	A	N/A	68, 69
				TK23	B	endogenous	68
tuberculosinol (**S76**)/<br>1-tuberculosinyladenosinec (**S77**)	O50406/<br>P9WJ61	Rv3377c/<br>Rv3378c	*Mycobacterium tuberculosis*	BL21(DE3)	A	N/A	70-73

[a]terpenes labeled with asterisk have unclear absolute stereochemistry, and those labeled with pound signs are the product of UbiA-type terpene cyclases; [b]BL21(DE3), BW25113, C41(DE3), C43(DE3), JM109, JM109(DE3), M15/pREP4, pLysS [BL21(DE3)pLysS], and Rossetta2(DE3) are *Escherichia coli* strains. SUKA16, SUKA17, and SUKA22 are *Streptomyces avermitilis* strains. TK21 and TK23 are *Streptomyces lividans* strains. NB: *Nicotiana benthaniana*. XD: *Xanthophyllomyces dendrorhous*. WT: terpenes are detected in the headspace of wild-type strains, and the assignment of terpene cyclase was based on analysis of genomic sequence and exclusion of other common terpene cyclases. [c]A: *in vitro*, B: *in vivo* studies; [d]several products were produced; [e]requires two enzymes: methyltransferase / cyclase; [f]accession numbers and protein names in the order of type II diterpene synthase / type I diterpene synthase.

Table S2: Functional characterization of bacterial tailoring enzymes for terpene modification.

Uniprot #	Name	Class[a]	Strain	Host[b]	Study[c]	Substrate[d]	Reaction	Ref
**Albaflavenone (S78, 35)**								
Q9K498	CYP107A1 sco5223	CYP	*Streptomyces coelicolor*	BL21(DE3)	A	*epi*-isozizaene (**S34, 33**)	hydroxylation-oxidation	74, 75
Q81IV2	CYP107A2 sav3031	CYP	*Streptomyces avermitilis*	SUKA16	B	*epi*-isozizaene (**S34, 33**)	hydroxylation-oxidation[e]	35

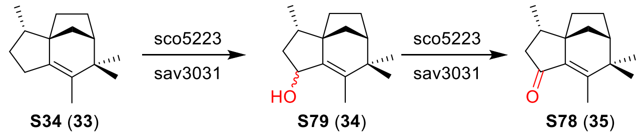

Uniprot #	Name	Class[a]	Strain	Host[b]	Study[c]	Substrate[d]	Reaction	Ref
**Pentalenolactone** (**S80**, **32**)								
Q82IY3/ E3VWK7/ E3VWJ1	PtlI/ PenI/ PntI[f]	CYP	*Streptomyces avermitilis/ Streptomyces exfoliatus/ Streptomyces arenae*	BL21(DE3)	A	pentalenene (**S37**)	4e-oxidation[g]	76, 77
Q82IZ1/ E3VWK0/ E3VWI4	PtlH/ PenH/ PntH[f]	AKG	as above	BL21(DE3)	A	1-deoxypentaleniate (**S81**)	hydroxylation	77-79
Q82IY9 E3VWK2/ E3VWI6	PtlF/ PenF/ PntF[f]	DH	as above	BL21(DE3)	A	**S82**	2e-oxidation	77, 80
Q82IY8	PtlE	FMO	*Streptomyces avermitilis*	BL21(DE3)	A	**S83**	Baeye-Villiger oxidation	81
Q82IY7	PtlD	AKG	*Streptomyces avermitilis*	BL21(DE3)	A	neopentalenolactone D (**S84**)	dehydrogenation	77
E3VWK3/ E3VWI7	PenE/ PntE	FMO	*Streptomyces exfoliatus/ Streptomyces arenae*	BL21(DE3)	A	**S83**	Baeye-Villiger oxidation	77
E3VWK4/ E3VWI8	PenD/ PntD	AKG	as above	BL21(DE3)	A	pentalenolactone D (**S86**)	dehydrogenation-epoxidation	77
E3VWJ9/ E3VWI3	PenM/ PntM	CYP	as above	BL21(DE3)	A	pentalenolactone F (**S87**, **31**)	rearrangement	82

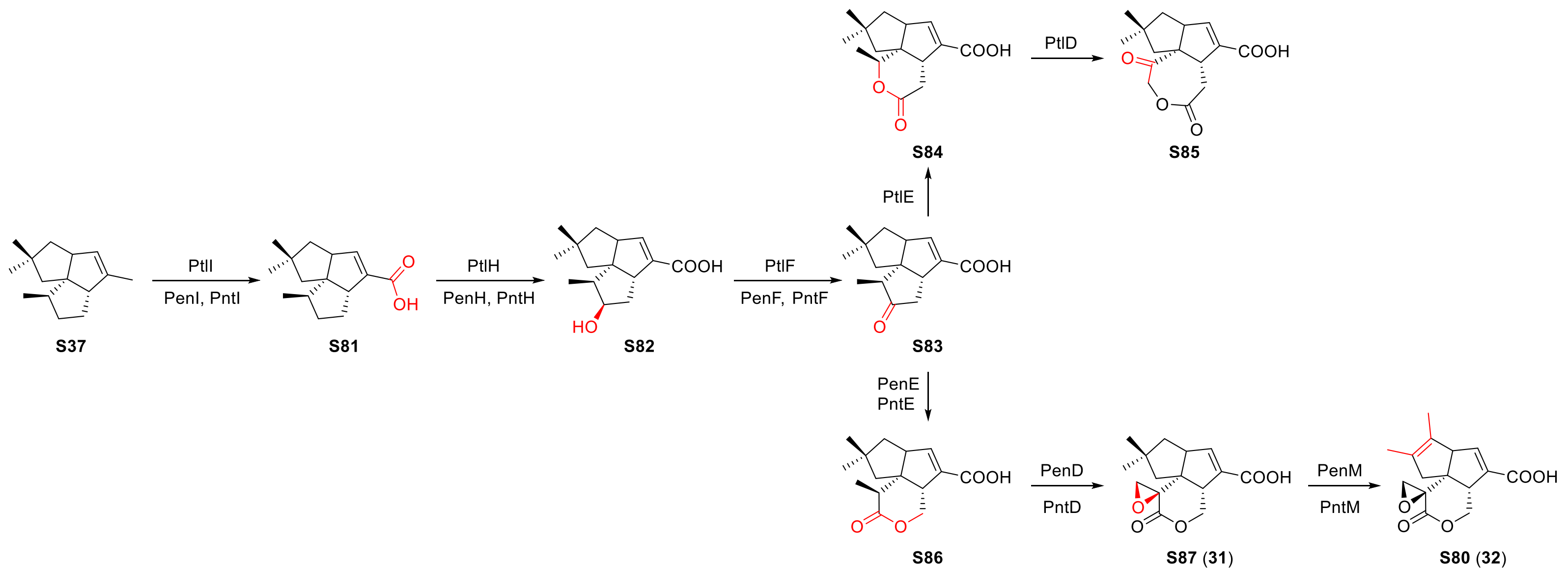

Cyclooctatin (S88, 25)

C9K1X6	CotB3	CYP	*Streptomyces melanosporofaciens*	*S. albus*	B	cyclooctat-9-en-7-ol (**S50**, **23**)	hydroxylation	48
C9K1X7	CotB4	CYP	*Streptomyces melanosporofaciens*	*S. albus*	B	**S89** (**24**)	hydroxylation	48

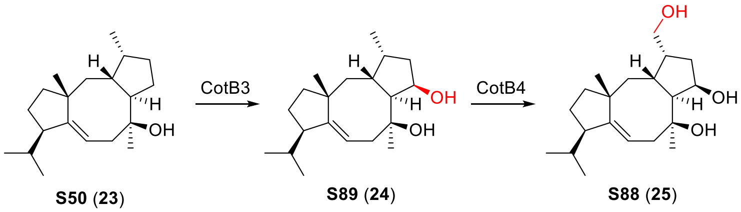

Cyslabdan (S90)

A0A0H5B5M5	CldC	CYP	*Streptomyces cyslabdanicus*	SUKA22	C	biformene (**S69**)	hydroxylation-epoxidation	60

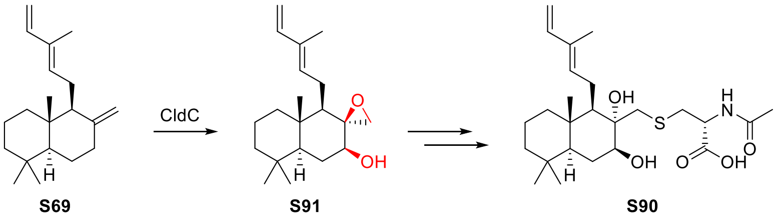

Gibberellinsh

P55540/ Q59205	CYP117A2	CYP	*Sinorhizobium fredii/ Bradyrhizobium diazoefficiens*	SM	C	*ent*-kaurene (**S72**, **16**)	6e-oxidation	83
P55543/ Q59204	CYP114	CYP	as above	SM	C	*ent*-kaurenoic acid (**S92**, **26**)	hydroxylation/ ring contraction	83
P55541/ Q45219	SDR	DH	as above	SM	C	**S93** (**28**)	2e-oxidation	83
P55544/ Q59203	CYP112A2	CYP	as above	SM	C	gibberellin A12 (**S94**)	demethylation-lactonization	83
UPI00040E8C18/ Q98A71	SMCYP115/ MlCYP115	CYP	*Sinorhizobium meliloti* / *Mesorhizobium japonicum*	SM	C	gibberellin A9 (**S95**)	hydroxylation	84

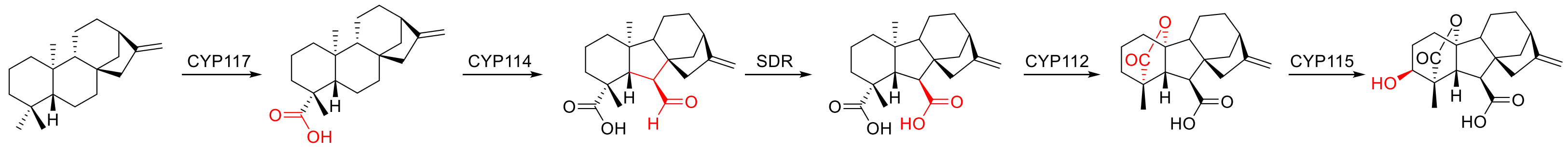

S72 (**16**) **S92** (**26**) **S93** (**28**) **S94** **S95** **S96**

Isopimara-8,15-dien-19-ol (**S97**)

A8M707	CYP1051A1 sare_1286	CYP	*Salinispora arenicola*	C43(DE3)	B	isopimara-8,15-diene (**S70**)	hydroxylation	62

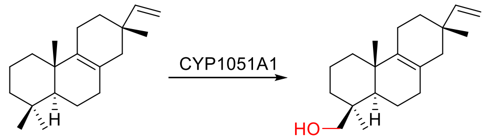

S70 **S97**

(+)-*O*-methylkolavelool (**S98**)

A9AWD7	Haur_2147	MT	*Herpetosiphon aurantiacus*	BL21(DE3)	A	(+)-kolavelool (**S73**)	methylation	65

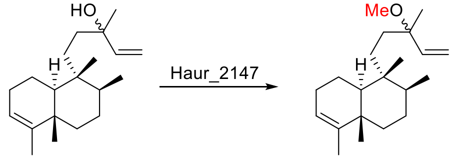

S73 **S98**

Platensimycin (**S99**) / **Platencin** (**S100**)

DBL2V0	PtmO5	CYP	*Streptomyces platensis*	BL21(DE3)	A	**S101** (**29**)	hydroxylation/ ether formation[i]	59, 85
DBL2U7	PtmO3	AKG	as above	BL21(DE3)	A/B	**S102** (**30**), **S109**	hydroxylation	86
DBL2V2	PtmO6	AKG	as above	BL21(DE3)	A/B	**S103**, **S110**	hydroxylation	86
DBL2W5	PtmO8	DH	as above	BL21(DE3)	A/B	**S104**, **S111**	oxidation	86
A0A0A0UXJ2	PtmO1	DH	as above	BL21(DE3)	A/B	**S105**, **S112**	reduction	86
DBL2T7	PtmA1	CoAS	as above	mutant	B	**S106**, **S113**	adenylation	87
DBL2U1	PtmA2	CoAS	as above	BL21(DE3)	A/B	**S107**, **S114**	ligation	87
DBL2W0	PtmC	NAT	as above	mutant	B	**S108**, **S115**	ligation	88

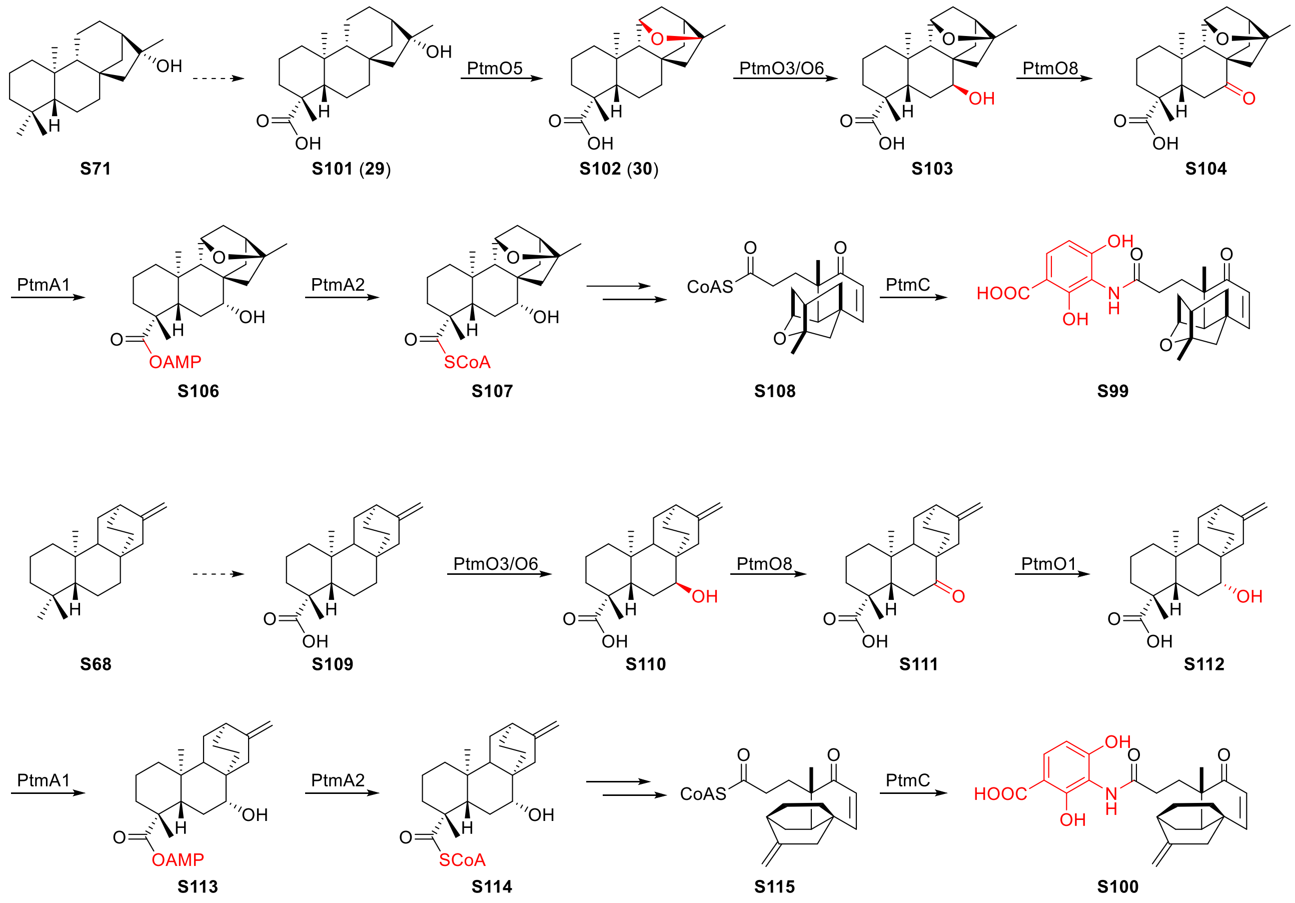

Others

P14779	$P450_{BM3}$	CYP	*Bacillus megaterium*	DH5α	A/C	*valencene (**S116**)	oxidation	89

	CYP102A1							

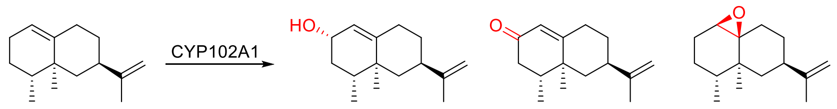

S116 **S117** **S118** **S119**

P18326	CYP105A1	CYP	*Streptomyces griseolus*	JM109	A/C	*abietic acid (**S120**)	hydroxylation	90, 91
				BM	C			90
				JM109	A/C	*dehydroabietic acid (**S121**)	hydroxylation	90
				BM	C			90
				JM109	A/C	*isopimaric acid (**S122**)	epoxidation	90
				BM	C			90

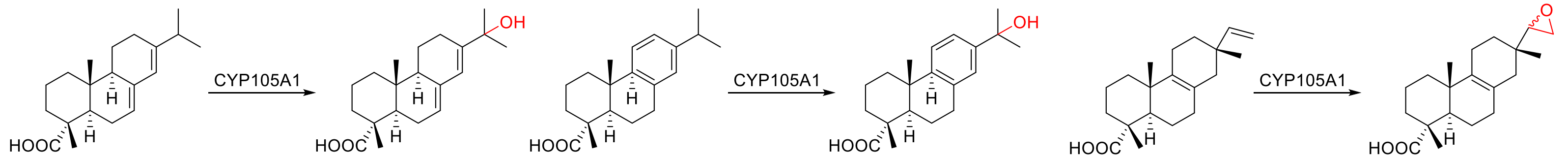

S120 **S123** **S121** **S124** **S122** **S125**

Q825I8	CYP105D7 sav7469	CYP	*S. avermitilis*	SUKA13	B	*1-deoxypentalenate (**S81**)	hydroxylation	92
				BL21(DE3)	A			92

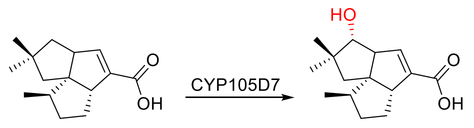

S81 **S126**

Q06069	CYP106A2	CYP	*Bacillus megaterium*	BL21(DE3)	A	*abietic acid (**S120**)	hydroxylation	93
				BM	C			

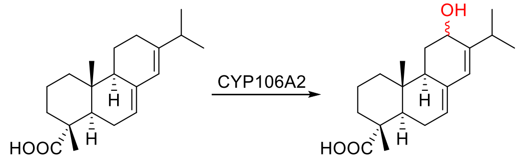

S120 **S127**

U5U1Z3	CYP109B1	CYP	*Bacillus subtilis*	BL21(DE3)	A/C	*valencene (**S116**)	hydroxylation/ oxidation	94

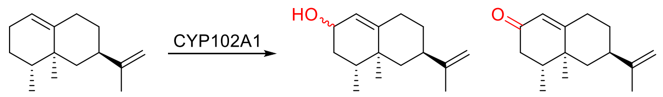

S116 ***rac*-S117** **S118**

Q8YN84	CYP110C1	CYP	*Nostoc punctiforme*	JM109 / BL21(DE3)	B	germacrene A (**S26**, **13**)	epoxidation-cyclization	23

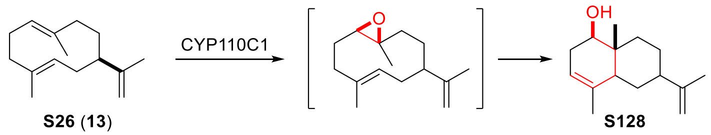

Q8YMU4	CYP110E1	CYP	*Nostoc sp.* PCC 7120	BL21(DE3)	C	*2,3-dihydrozerumbone (**S130**)[j]	hydroxylation	15

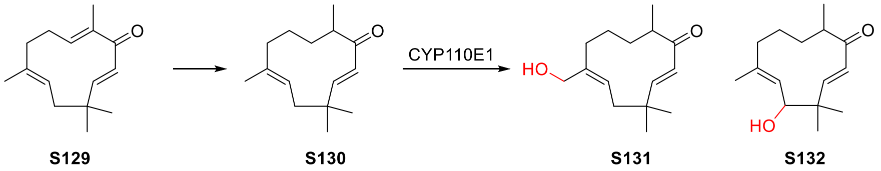

Q47KL3	CYP154E1	CYP	*Thermobifida fusca*	BL21(DE3)	A	*(+)-nootkatone (**S118**)	hydroxylation	95
						*(+)-nootkatol (**S117**)	oxidation	95

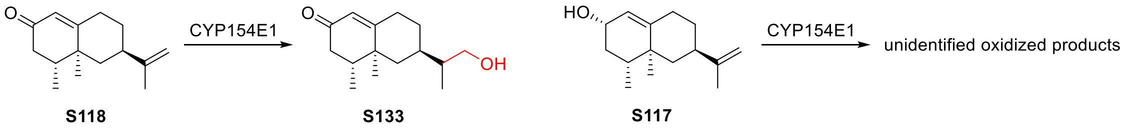

A9FDB7	CYP260A1 sce1588	CYP	*Sorangium cellulosum* So ce56	BL21(DE3)	A/C	*(+)-nootkatone (**S118**)	oxidation	96, 97
					C	*zerumbone (**S129**)	oxidation	97
					C	*(+)-3(15)-cedren-4-ol (**S134**)	oxidation	97

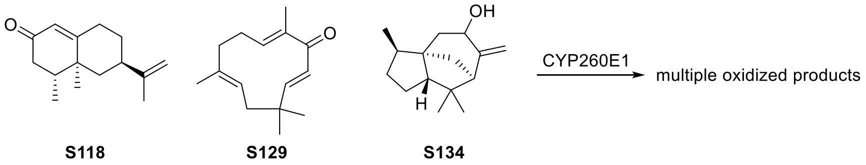

A9FZ85	CYP264B1 sce8851	CYP	*Sorangium cellulosum* So ce56	C43(DE3)	A/B/C	(+)-eremophilene (**S20**)	hydroxylation	15, 97
				C43(DE3) / BL21(DE3)	C	*(+)-nootkatone (**S118**)	hydroxylation	97
					C	*zerumbone (**S129**)	hydroxylation	97
					A/C	*α-humulene (**S135**)	hydroxylation	97, 98
					C	*β-caryophyllene (**S13**)	hydroxylation	97
					C	*α-cedrene (**S136**)	hydroxylation	97
					A/C	*(+)-α-longipinene (**S137**)	hydroxylation	98
					A/C	*(-)-isolongifolene (**S138**)	4e-oxidation	98

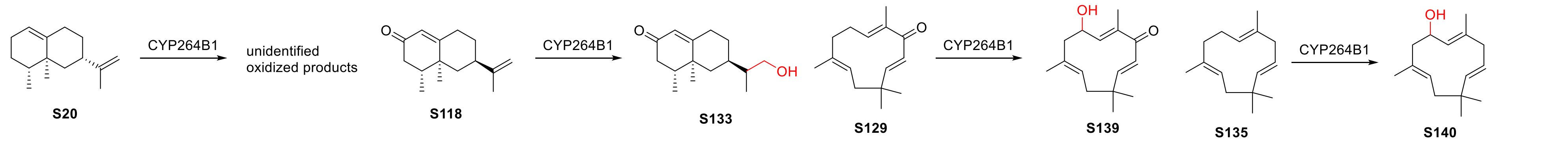

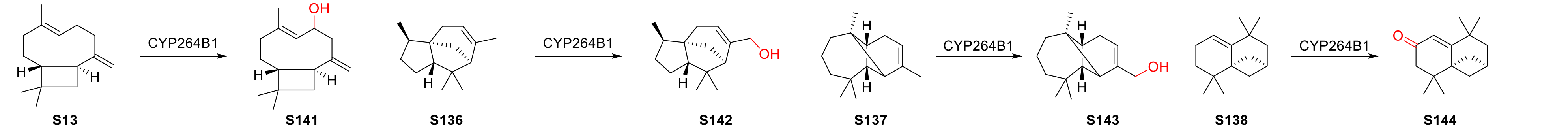

A9ERX9	CYP267B1	CYP	*Sorangium cellulosum* So ce56	C43(DE3)	A	*(+)-T-muurolol (**S35**)	hydroxylation-oxidation	99
					A	*(+)-isodauc-8-en-11-ol (**S18**)	epoxidation-hydroxylation	99

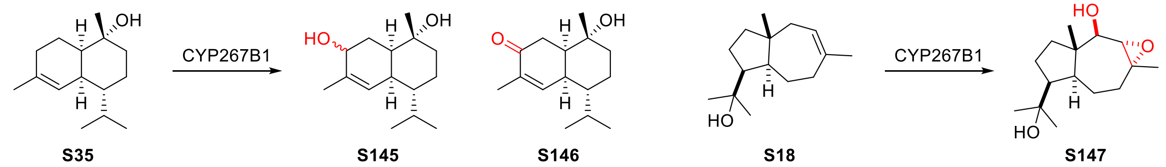

[a]AKG: α-ketoglutarate-dependent non-heme iron enzyme, CoAS: acyl-CoA synthetase, CYP: cytochrome P450, DH: NAD(P)$^+$-dependent dehydrogenase, FMO: Flavin-dependent monooxygenase, MT: methyltransferase, NAT: *N*-acetyl transferase; [b]BL21(DE3), C43(DE3), DH5α, JM109: *E. coli* strains. SUKA13, SUKA16, SUKA22: *S. avermitilis* strains. BM: *Bacillus megaterium* MS941. SM: *Sinorhizobium meliloti* 1021. [c]A: in vitro, B: in vivo: the heterologous host expresses both cyclase and tailoring enzymes, or gene deletion / complementation experiments in the producing strains, C: growing cells or resting cells expressing tailoring enzymes are fed with substrate directly; [d]substrates marked with asterisk are not produced by any enzyme(s) encoded nearby the tailoring enzymes; [e]epoxidation took place when expressed in *S. avermitilis*, which might come from the action of endogenous P450 of the heterologous host. Also see Figure 8 in the main text; [f]only Ptl enzymes were experimentally verifies. Pen and Pnt enzymes' function were implied by sequence homology. [g]6e-oxidaion was expected, but *in vitro* reaction only catalyzed 4e-oxidation; [h]*Sinorhizobium fredii* enzymes were studied by feeding substrates to *Sinorhizobium meliloti* 1021 expressing enzymes of interest. Substrates were also fed to *S. fredii / Bradyrhizobium diazoefficiens* deletion strains to map out the biosynthetic pathway; [i]in the in vitro studies, HCl was required to trigger the ether ring formation; [j]it was claimed to come from the reduction of zerumbone (the fed substrate, **S128**) by endogenous enzymes of *E. coli*.

References

1. Yamada, Y.; Kuzuyama, T.; Komatsu, M.; Shin-ya, K.; Omura, S.; Cane, D. E.; Ikeda, H., Terpene synthases are widely distributed in bacteria. *Proceedings of the National Academy of Sciences* **2015,** *112*, 857-862.

2. Rabe, P.; Dickschat, J. S., Rapid chemical characterization of bacterial terpene synthases. *Angewandte Chemie - International Edition* **2013,** *52*, 1810-1812.

3. Rabe, P.; Schmitz, T.; Dickschat, J. S., Mechanistic investigations on six bacterial terpene cyclases. *Beilstein Journal of Organic Chemistry* **2016,** *12*, 1839-1850.

4. Rinkel, J.; Rabe, P.; Garbeva, P.; Dickschat, J. S., Lessons from 1,3-Hydride Shifts in Sesquiterpene Cyclizations. *Angewandte Chemie International Edition* **2016,** *55*, 13593-13596.

5. Chou, W. K. W.; Fanizza, I.; Uchiyama, T.; Komatsu, M.; Ikeda, H.; Cane, D. E., Genome Mining in Streptomyces avermitilis : Cloning and Characterization of SAV_76, the Synthase for a New Sesquiterpene, Avermitilol. *Journal of the American Chemical Society* **2010,** *132*, 8850-8851.

6. Schifrin, A.; Khatri, Y.; Kirsch, P.; Thiel, V.; Schulz, S.; Bernhardt, R., A single terpene synthase is responsible for a wide variety of sesquiterpenes in Sorangium cellulosum Soce56. *Organic & Biomolecular Chemistry* **2016,** *14*, 3385-3393.

7. Nakano, C.; Kudo, F.; Eguchi, T.; Ohnishi, Y., Genome Mining Reveals Two Novel Bacterial Sesquiterpene Cyclases: (−)-Germacradien-4-ol and (−)-epi-α-Bisabolol Synthases from Streptomyces citricolor. *ChemBioChem* **2011,** *12*, 2271-2275.

8. Hu, Y.; Chou, W. K. W.; Hopson, R.; Cane, D. E., Genome Mining in Streptomyces clavuligerus: Expression and Biochemical Characterization of Two New Cryptic Sesquiterpene Synthases. *Chemistry & Biology* **2011,** *18*, 32-37.

9. Nakano, C.; Horinouchi, S.; Ohnishi, Y., Characterization of a Novel Sesquiterpene Cyclase Involved in (+)-Caryolan-1-ol Biosynthesis in Streptomyces griseus. *Journal of Biological Chemistry* **2011,** *286*, 27980-27987.

10. Dickschat, J. S.; Pahirulzaman, K. A. K.; Rabe, P.; Klapschinski, T. A., An Improved technique for the rapid chemical characterisation of bacterial terpene cyclases. *ChemBioChem* **2014,** *15*, 810-814.

11. Rabe, P.; Pahirulzaman, K. A. K.; Dickschat, J. S., Structures and biosynthesis of corvol ethers - Sesquiterpenes from the actinomycete kitasatospora setae. *Angewandte Chemie - International Edition* **2015,** *54*, 6041-6045.

12. Rabe, P.; Dickschat, J. S., The EIMS fragmentation mechanisms of the sesquiterpenes corvol ethers A and B, epi -cubebol and isodauc-8-en-11-ol. *Beilstein Journal of Organic Chemistry* **2016,** *12*, 1380-1394.

13. Rabe, P.; Rinkel, J.; Klapschinski, T. A.; Barra, L.; Dickschat, J. S., A method for investigating the stereochemical course of terpene cyclisations. *Organic & Biomolecular Chemistry* **2016,** *14*, 158-164.

14. Nakano, C.; Tezuka, T.; Horinouchi, S.; Ohnishi, Y., Identification of the SGR6065 gene product as a sesquiterpene cyclase involved in (+)-epicubenol biosynthesis in Streptomyces griseus. *The Journal of Antibiotics* **2012,** *65*, 551-558.

15. Schifrin, A.; Ly, T. T. B.; Günnewich, N.; Zapp, J.; Thiel, V.; Schulz, S.; Hannemann, F.; Khatri, Y.; Bernhardt, R., Characterization of the Gene Cluster CYP264B1- geo A from Sorangium cellulosum So ce56: Biosynthesis of (+)-Eremophilene and Its Hydroxylation. *ChemBioChem* **2015,** *16*, 337-344.

16. Rabe, P.; Barra, L.; Rinkel, J.; Riclea, R.; Citron, C. A.; Klapschinski, T. A.; Janusko, A.; Dickschat, J. S., Conformational Analysis, Thermal Rearrangement, and EI-MS Fragmentation Mechanism of (1(10)E,4E,6S,7R)-Germacradien-6-ol by13C-Labeling Experiments. *Angewandte Chemie - International Edition* **2015,** *54*, 13448-13451.

17. Jiang, J.; He, X.; Cane, D. E., Geosmin Biosynthesis. Streptomyces coelicolor Germacradienol/Germacrene D Synthase Converts Farnesyl Diphosphate to Geosmin. *Journal of the American Chemical Society* **2006,** *128*, 8128-8129.

18. Jiang, J.; Cane, D. E., Geosmin Biosynthesis. Mechanism of the Fragmentation−Rearrangement in the Conversion of Germacradienol to Geosmin. *Journal of the American Chemical Society* **2008,** *130*, 428-429.

19. Cane, D. E.; Watt, R. M., Expression and mechanistic analysis of a germacradienol synthase from Streptomyces coelicolor implicated in geosmin biosynthesis. *Proceedings of the National Academy of Sciences* **2003,** *100*, 1547-1551.

20. Jiang, J.; He, X.; Cane, D. E., Biosynthesis of the earthy odorant geosmin by a bifunctional Streptomyces coelicolor enzyme. *Nature Chemical Biology* **2007,** *3*, 711-715.

21. Harris, G. G.; Lombardi, P. M.; Pemberton, T. A.; Matsui, T.; Weiss, T. M.; Cole, K. E.; Köksal, M.; Murphy, F. V.; Vedula, L. S.; Chou, W. K. W.; Cane, D. E.; Christianson, D. W., Structural Studies of Geosmin Synthase, a Bifunctional Sesquiterpene Synthase with αα Domain Architecture That Catalyzes a Unique Cyclization–Fragmentation Reaction Sequence. *Biochemistry* **2015,** *54*, 7142-7155.

22. Cane, D. E.; He, X.; Kobayashi, S.; Ōmura, S.; Ikeda, H., Geosmin Biosynthesis in Streptomyces avermitilis. Molecular Cloning, Expression and Mechanistic Study of the Germacradienol/Geosmin Synthase. *The Journal of Antibiotics* **2006,** *59*, 471-479.

23. Agger, S. A.; Lopez-Gallego, F.; Hoye, T. R.; Schmidt-Dannert, C., Identification of sesquiterpene synthases from Nostoc punctiforme PCC 73102 and Nostoc sp. strain PCC 7120. *Journal of Bacteriology* **2008,** *190*, 6084-6096.

24. Jiang, J.; Saint, C. P.; Cane, D. E.; Monis, P. T., Isolation and Characterization of the Gene Associated with Geosmin Production in Cyanobacteria. *Environmental Science & Technology* **2008,** *42*, 8027-8032.

25. Ghimire, G. P.; Oh, T. J.; Lee, H. C.; Kim, B. G.; Sohng, J. K., Cloning and functional characterization of the germacradienol synthase (spterp13) from Streptomyces peucetius ATCC 27952. *Journal of Microbiology and Biotechnology* **2008,** *18*, 1216-1220.

26. Baer, P.; Rabe, P.; Citron, C. A.; De Oliveira Mann, C. C.; Kaufmann, N.; Groll, M.; Dickschat, J. S., Hedycaryol synthase in complex with nerolidol reveals terpene cyclase mechanism. *ChemBioChem* **2014,** *15*, 213-216.

27. Riclea, R.; Citron, C. A.; Rinkel, J.; Dickschat, J. S., Identification of isoafricanol and its terpene cyclase in Streptomyces violaceusniger using CLSA-NMR. *Chemical Communications* **2014,** *50*, 4228-4230.

28. Rabe, P.; Samborskyy, M.; Leadlay, P. F.; Dickschat, J. S., Isoafricanol synthase from Streptomyces malaysiensis. *Organic & Biomolecular Chemistry* **2017,** *15*, 2353-2358.

29. Yamada, Y.; Arima, S.; Nagamitsu, T.; Johmoto, K.; Uekusa, H.; Eguchi, T.; Shin-Ya, K.; Cane, D. E.; Ikeda, H., Novel terpenes generated by heterologous expression of bacterial terpene synthase genes in an engineered Streptomyces host. *Journal of Antibiotics* **2015,** *68*, 385-394.

30. Chow, J.-Y.; Tian, B.-X.; Ramamoorthy, G.; Hillerich, B. S.; Seidel, R. D.; Almo, S. C.; Jacobson, M. P.; Poulter, C. D., Computational-guided discovery and characterization of a sesquiterpene synthase from Streptomyces clavuligerus. *Proceedings of the National Academy of Sciences* **2015,** *112*, 5661-5666.

31. Lin, X.; Hopson, R.; Cane, D. E., Genome mining in Streptomyces coelicolor: Molecular cloning and characterization of a new sesquiterpene synthase. *Journal of the American Chemical Society* **2006,** *128*, 6022-6023.

32. Lin, X.; Cane, D. E., Biosynthesis of the Sesquiterpene Antibiotic Albaflavenone in Streptomyces coelicolor . Mechanism and Stereochemistry of the Enzymatic Formation of Epi-isozizaene. *Journal of the American Chemical Society* **2009,** *131*, 6332-6333.

33. Aaron, J. A.; Lin, X.; Cane, D. E.; Christianson, D. W., Structure of Epi-Isozizaene Synthase from Streptomyces coelicolor A3(2), a Platform for New Terpenoid Cyclization Templates. *Biochemistry* **2010,** *49*, 1787-1797.

34. Li, R.; Chou, W. K. W.; Himmelberger, J. A.; Litwin, K. M.; Harris, G. G.; Cane, D. E.; Christianson, D. W., Reprogramming the Chemodiversity of Terpenoid Cyclization by Remolding the Active Site Contour of epi -Isozizaene Synthase. *Biochemistry* **2014,** *53*, 1155-1168.

35. Takamatsu, S.; Lin, X.; Nara, A.; Komatsu, M.; Cane, D. E.; Ikeda, H., Characterization of a silent sesquiterpenoid biosynthetic pathway in Streptomyces avermitilis controlling epi-isozizaene albaflavenone biosynthesis and isolation of a new oxidized epi-isozizaene metabolite. *Microbial Biotechnology* **2011,** *4*, 184-191.

36. Rinkel, J.; Rabe, P.; Dickschat, J. S., The EI-MS Fragmentation Mechanisms of Bacterial Sesquiterpenes and Diterpenes. *European Journal of Organic Chemistry* **2019,** *2019*, 351-359.

37. Cane, D. E.; Sohng, J. K.; Lamberson, C. R.; Rudnicki, S. M.; Wu, Z.; Lloyd, M. D.; Oliver, J. S.; Hubbard, B. R., Pentalenene Synthase. Purification, Molecular Cloning, Sequencing, and High–Level Expression in Escherichia coli of a Terpenoid Cyclase from Streptomyces UC5319. *Biochemistry* **1994,** *33*, 5846-5857.

38. Lesburg, C. A., Crystal Structure of Pentalenene Synthase: Mechanistic Insights on Terpenoid Cyclization Reactions in Biology. *Science* **1997,** *277*, 1820-1824.

39. Melillo, E.; Muntendam, R.; Quax, W. J.; Kayser, O., Heterologous expression of pentalenene synthase (PSS) from Streptomyces UC5319 in Xanthophyllomyces dendrorhous. *Journal of Biotechnology* **2012,** *161*, 302-307.

40. Tetzlaff, C. N.; You, Z.; Cane, D. E.; Takamatsu, S.; Omura, S.; Ikeda, H., A Gene Cluster for Biosynthesis of the Sesquiterpenoid Antibiotic Pentalenolactone in Streptomyces avermitilis †. *Biochemistry* **2006,** *45*, 6179-6186.

41. Klapschinski, T. A.; Rabe, P.; Dickschat, J. S., Pristinol, a Sesquiterpene Alcohol with an Unusual Skeleton from Streptomyces pristinaespiralis. *Angewandte Chemie International Edition* **2016,** *55*, 10141-10144.

42. Rabe, P.; Citron, C. A.; Dickschat, J. S., Volatile Terpenes from Actinomycetes: A Biosynthetic Study Correlating Chemical Analyses to Genome Data. *ChemBioChem* **2013,** *14*, 2345-2354.

43. von Reuss, S.; Domik, D.; Lemfack, M. C.; Magnus, N.; Kai, M.; Weise, T.; Piechulla, B., Sodorifen Biosynthesis in the Rhizobacterium Serratia plymuthica Involves Methylation and Cyclization of MEP-Derived Farnesyl Pyrophosphate by a SAM-Dependent C -Methyltransferase. *Journal of the American Chemical Society* **2018,** *140*, 11855-11862.

44. Duell, E. R.; D'Agostino, P. M.; Shapiro, N.; Woyke, T.; Fuchs, T. M.; Gulder, T. A. M., Direct pathway cloning of the sodorifen biosynthetic gene cluster and recombinant generation of its product in E. coli. *Microbial Cell Factories* **2019,** *18*, 32-32.

45. Yang, Y.-l.; Zhang, S.; Ma, K.; Xu, Y.; Tao, Q.; Chen, Y.; Chen, J.; Guo, S.; Ren, J.; Wang, W.; Tao, Y.; Yin, W.-B.; Liu, H., Discovery and Characterization of a New Family of Diterpene Cyclases in Bacteria and Fungi. *Angewandte Chemie International Edition* **2017,** *56*, 4749-4752.

46. Lauterbach, L.; Rinkel, J.; Dickschat, J. S., Two Bacterial Diterpene Synthases from Allokutzneria albata Produce Bonnadiene, Phomopsene, and Allokutznerene. *Angewandte Chemie International Edition* **2018,** *57*, 8280-8283.

47. Rinkel, J.; Lauterbach, L.; Rabe, P.; Dickschat, J. S., Two Diterpene Synthases for Spiroalbatene and Cembrene A from Allokutzneria albata. *Angewandte Chemie International Edition* **2018,** *57*, 3238-3241.

48. Kim, S. Y.; Zhao, P.; Igarashi, M.; Sawa, R.; Tomita, T.; Nishiyama, M.; Kuzuyama, T., Cloning and Heterologous Expression of the Cyclooctatin Biosynthetic Gene Cluster Afford a Diterpene Cyclase and Two P450 Hydroxylases. *Chemistry and Biology* **2009,** *16*, 736-743.

49. Meguro, A.; Motoyoshi, Y.; Teramoto, K.; Ueda, S.; Totsuka, Y.; Ando, Y.; Tomita, T.; Kim, S.-Y.; Kimura, T.; Igarashi, M.; Sawa, R.; Shinada, T.; Nishiyama, M.; Kuzuyama, T., An Unusual Terpene Cyclization Mechanism Involving a Carbon-Carbon Bond Rearrangement. *Angewandte Chemie International Edition* **2015,** *54*, 4353-4356.

50. Görner, C.; Häuslein, I.; Schrepfer, P.; Eisenreich, W.; Brück, T., Targeted Engineering of Cyclooctat-9-en-7-ol Synthase: A Stereospecific Access to Two New Non-natural Fusicoccane-Type Diterpenes. *ChemCatChem* **2013,** *5*, 3289-3298.

51. Tomita, T.; Kim, S.-Y.; Teramoto, K.; Meguro, A.; Ozaki, T.; Yoshida, A.; Motoyoshi, Y.; Mori, N.; Ishigami, K.; Watanabe, H.; Nishiyama, M.; Kuzuyama, T., Structural Insights into the CotB2-Catalyzed Cyclization of Geranylgeranyl Diphosphate to the Diterpene Cyclooctat-9-en-7-ol. *ACS Chemical Biology* **2017,** *12*, 1621-1628.

52. Driller, R.; Janke, S.; Fuchs, M.; Warner, E.; Mhashal, A. R.; Major, D. T.; Christmann, M.; Brück, T.; Loll, B., Towards a comprehensive understanding of the structural dynamics of a bacterial diterpene synthase during catalysis. *Nature Communications* **2018,** *9*, 3971-3971.

53. Rinkel, J.; Rabe, P.; Chen, X.; Köllner, T. G.; Chen, F.; Dickschat, J. S., Mechanisms of the Diterpene Cyclases β-Pinacene Synthase from Dictyostelium discoideum and Hydropyrene Synthase from Streptomyces clavuligerus. *Chemistry - A European Journal* **2017,** *23*, 10501-10505.

54. Dickschat, J. S.; Rinkel, J.; Rabe, P.; Beyraghdar Kashkooli, A.; Bouwmeester, H. J., 18-Hydroxydolabella-3,7-diene synthase – a diterpene synthase from Chitinophaga pinensis. *Beilstein Journal of Organic Chemistry* **2017,** *13*, 1770-1780.

55. Meguro, A.; Tomita, T.; Nishiyama, M.; Kuzuyama, T., Identification and Characterization of Bacterial Diterpene Cyclases that Synthesize the Cembrane Skeleton. *ChemBioChem* **2013,** *14*, 316-321.

56. Rinkel, J.; Lauterbach, L.; Dickschat, J. S., Spata-13,17-diene Synthase-An Enzyme with Sesqui-, Di-, and Sesterterpene Synthase Activity from Streptomyces xinghaiensis. *Angewandte Chemie International Edition* **2017,** *56*, 16385-16389.

57. Rabe, P.; Rinkel, J.; Dolja, E.; Schmitz, T.; Nubbemeyer, B.; Luu, T. H.; Dickschat, J. S., Mechanistic Investigations of Two Bacterial Diterpene Cyclases: Spiroviolene Synthase and Tsukubadiene Synthase. *Angewandte Chemie International Edition* **2017,** *56*, 2776-2779.

58. Rudolf, J. D.; Dong, L.-B.; Cao, H.; Hatzos-Skintges, C.; Osipiuk, J.; Endres, M.; Chang, C.-Y.; Ma, M.; Babnigg, G.; Joachimiak, A.; Phillips, G. N.; Shen, B., Structure of the ent -Copalyl Diphosphate Synthase PtmT2 from Streptomyces platensis CB00739, a Bacterial Type II Diterpene Synthase. *Journal of the American Chemical Society* **2016,** *138*, 10905-10915.

59. Rudolf, J. D.; Dong, L.-B.; Manoogian, K.; Shen, B., Biosynthetic Origin of the Ether Ring in Platensimycin. *Journal of the American Chemical Society* **2016,** *138*, 16711-16721.

60. Ikeda, H.; Shin-ya, K.; Nagamitsu, T.; Tomoda, H., Biosynthesis of mercapturic acid derivative of the labdane-type diterpene, cyslabdan that potentiates imipenem activity against methicillin-resistant Staphylococcus aureus: cyslabdan is generated by mycothiol-mediated xenobiotic detoxification. *Journal of Industrial Microbiology & Biotechnology* **2016,** *43*, 325-342.

61. Yamada, Y.; Komatsu, M.; Ikeda, H., Chemical diversity of labdane-type bicyclic diterpene biosynthesis in Actinomycetales microorganisms. *The Journal of Antibiotics* **2016,** *69*, 515-523.

62. Xu, M.; Hillwig, M. L.; Lane, A. L.; Tiernan, M. S.; Moore, B. S.; Peters, R. J., Characterization of an Orphan Diterpenoid Biosynthetic Operon from Salinispora arenicola. *Journal of Natural Products* **2014,** *77*, 2144-2147.

63. Morrone, D.; Chambers, J.; Lowry, L.; Kim, G.; Anterola, A.; Bender, K.; Peters, R. J., Gibberellin biosynthesis in bacteria: Separate ent-copalyl diphosphate and ent-kaurene synthases in Bradyrhizobium japonicum. *FEBS Letters* **2009,** *583*, 475-480.

64. Hershey, D. M.; Lu, X.; Zi, J.; Peters, R. J., Functional Conservation of the Capacity for ent-Kaurene Biosynthesis and an Associated Operon in Certain Rhizobia. *Journal of Bacteriology* **2014,** *196*, 100-106.

65. Nakano, C.; Oshima, M.; Kurashima, N.; Hoshino, T., Identification of a New Diterpene Biosynthetic Gene Cluster that Produces O -Methylkolavelool in Herpetosiphon aurantiacus. *ChemBioChem* **2015,** *16*, 772-781.

66. Ikeda, C.; Hayashi, Y.; Itoh, N.; Seto, H.; Dairi, T., Functional analysis of eubacterial ent-copalyl diphosphate synthase and pimara-9(11),15-diene synthase with unique primary sequences. *Journal of Biochemistry* **2007,** *141*, 37-45.

67. Kawasaki, T.; Kuzuyama, T.; Kuwamori, Y.; Matsuura, N.; Itoh, N.; Furihata, K.; Seto, H.; Dairi, T., Presence of Copalyl Diphosphate Synthase Gene in an Actinomycete Possessing the Mevalonate Pathway. *The Journal of Antibiotics* **2004,** *57*, 739-747.

68. Dairi, T.; Hamano, Y.; Kuzuyama, T.; Itoh, N.; Furihata, K.; Seto, H., Eubacterial diterpene cyclase genes essential for production of the isoprenoid antibiotic terpentecin. *Journal of Bacteriology* **2001,** *183*, 6085-6094.

69. Hamano, Y.; Kuzuyama, T.; Itoh, N.; Furihata, K.; Seto, H.; Dairi, T., Functional analysis of eubacterial diterpene cyclases responsible for biosynthesis of a diterpene antibiotic, terpentecin. *Journal of Biological Chemistry* **2002,** *277*, 37098-37104.

70. Nakano, C.; Okamura, T.; Sato, T.; Dairi, T.; Hoshino, T., Mycobacterium tuberculosis H37Rv3377c encodes the diterpene cyclase for producing the halimane skeleton. *Chemical Communications* **2005**, 1016-1016.

71. Nakano, C.; Hoshino, T., Characterization of the Rv3377c Gene Product, a Type-B Diterpene Cyclase, from the Mycobacterium tuberculosis H37 Genome. *ChemBioChem* **2009,** *10*, 2060-2071.

72. Nakano, C.; Ootsuka, T.; Takayama, K.; Mitsui, T.; Sato, T.; Hoshino, T., Characterization of the Rv3378c Gene Product, a New Diterpene Synthase for Producing Tuberculosinol and (13 R , S)-Isotuberculosinol (Nosyberkol), from the Mycobacterium tuberculosis H37Rv Genome. *Bioscience, Biotechnology, and Biochemistry* **2011,** *75*, 75-81.

73. Layre, E.; Lee, H. J.; Young, D. C.; Jezek Martinot, A.; Buter, J.; Minnaard, A. J.; Annand, J. W.; Fortune, S. M.; Snider, B. B.; Matsunaga, I.; Rubin, E. J.; Alber, T.; Moody, D. B., Molecular profiling of Mycobacterium tuberculosis identifies tuberculosinyl nucleoside products of the virulence-associated enzyme Rv3378c. *Proceedings of the National Academy of Sciences* **2014,** *111*, 2978-2983.

74. Zhao, B.; Lin, X.; Lei, L.; Lamb, D. C.; Kelly, S. L.; Waterman, M. R.; Cane, D. E., Biosynthesis of the Sesquiterpene Antibiotic Albaflavenone in Streptomyces coelicolor A3(2). *Journal of Biological Chemistry* **2008,** *283*, 8183-8189.

75. Zhao, B.; Lei, L.; Vassylyev, D. G.; Lin, X.; Cane, D. E.; Kelly, S. L.; Yuan, H.; Lamb, D. C.; Waterman, M. R., Crystal Structure of Albaflavenone Monooxygenase Containing a Moonlighting Terpene Synthase Active Site. *Journal of Biological Chemistry* **2009,** *284*, 36711-36719.

76. Quaderer, R.; Omura, S.; Ikeda, H.; Cane, D. E., Pentalenolactone Biosynthesis. Molecular Cloning and Assignment of Biochemical Function to PtlI, a Cytochrome P450 of Streptomyces avermitilis. *Journal of the American Chemical Society* **2006,** *128*, 13036-13037.

77. Seo, M.-J.; Zhu, D.; Endo, S.; Ikeda, H.; Cane, D. E., Genome Mining in Streptomyces . Elucidation of the Role of Baeyer−Villiger Monooxygenases and Non-Heme Iron-Dependent Dehydrogenase/Oxygenases in the Final Steps of the Biosynthesis of Pentalenolactone and Neopentalenolactone. *Biochemistry* **2011,** *50*, 1739-1754.

78. You, Z.; Omura, S.; Ikeda, H.; Cane, D. E., Pentalenolactone Biosynthesis. Molecular Cloning and Assignment of Biochemical Function to PtlH, A Non-Heme Iron Dioxygenase of Streptomyces avermitilis. *Journal of the American Chemical Society* **2006,** *128*, 6566-6567.

79. You, Z.; Omura, S.; Ikeda, H.; Cane, D. E.; Jogl, G., Crystal Structure of the Non-heme Iron Dioxygenase PtlH in Pentalenolactone Biosynthesis. *Journal of Biological Chemistry* **2007,** *282*, 36552-36560.

80. You, Z.; Omura, S.; Ikeda, H.; Cane, D. E., Pentalenolactone biosynthesis: Molecular cloning and assignment of biochemical function to PtlF, a short-chain dehydrogenase from Streptomyces avermitilis, and identification of a new biosynthetic intermediate. *Archives of Biochemistry and Biophysics* **2007,** *459*, 233-240.

81. Jiang, J.; Tetzlaff, C. N.; Takamatsu, S.; Iwatsuki, M.; Komatsu, M.; Ikeda, H.; Cane, D. E., Genome Mining in Streptomyces avermitilis : A Biochemical Baeyer−Villiger Reaction and Discovery of a New Branch of the Pentalenolactone Family Tree. *Biochemistry* **2009,** *48*, 6431-6440.

82. Zhu, D.; Seo, M.-J.; Ikeda, H.; Cane, D. E., Genome Mining in Streptomyces . Discovery of an Unprecedented P450-Catalyzed Oxidative Rearrangement That Is the Final Step in the Biosynthesis of Pentalenolactone. *Journal of the American Chemical Society* **2011,** *133*, 2128-2131.

83. Nett, R. S.; Montanares, M.; Marcassa, A.; Lu, X.; Nagel, R.; Charles, T. C.; Hedden, P.; Rojas, M. C.; Peters, R. J., Elucidation of gibberellin biosynthesis in bacteria reveals convergent evolution. *Nature Chemical Biology* **2017,** *13*, 69-74.

84. Nett, R. S.; Contreras, T.; Peters, R. J., Characterization of CYP115 As a Gibberellin 3-Oxidase Indicates That Certain Rhizobia Can Produce Bioactive Gibberellin A 4. *ACS Chemical Biology* **2017,** *12*, 912-917.

85. Rudolf, J. D.; Dong, L.-B.; Zhang, X.; Renata, H.; Shen, B., Cytochrome P450-Catalyzed Hydroxylation Initiating Ether Formation in Platensimycin Biosynthesis. *Journal of the American Chemical Society* **2018,** *140*, 12349-12353.

86. Dong, L.-B.; Zhang, X.; Rudolf, J. D.; Deng, M.-R.; Kalkreuter, E.; Cepeda, A. J.; Renata, H.; Shen, B., Cryptic and Stereospecific Hydroxylation, Oxidation, and Reduction in Platensimycin and Platencin Biosynthesis. *Journal of the American Chemical Society* **2019,** *141*, 4043-4050.

87. Wang, N.; Rudolf, J. D.; Dong, L.-B.; Osipiuk, J.; Hatzos-Skintges, C.; Endres, M.; Chang, C.-Y.; Babnigg, G.; Joachimiak, A.; Phillips, G. N.; Shen, B., Natural separation of the acyl-CoA ligase reaction results in a non-adenylating enzyme. *Nature Chemical Biology* **2018,** *14*, 730-737.

88. Dong, L.-B.; Rudolf, J. D.; Shen, B., A Mutasynthetic Library of Platensimycin and Platencin Analogues. *Organic Letters* **2016,** *18*, 4606-4609.

89. Sowden, R. J.; Yasmin, S.; Rees, N. H.; Bell, S. G.; Wong, L.-L., Biotransformation of the sesquiterpene (+)-valencene by cytochrome P450cam and P450BM-3. *Organic & Biomolecular Chemistry* **2005,** *3*, 57-57.

90. Janocha, S.; Zapp, J.; Hutter, M.; Kleser, M.; Bohlmann, J.; Bernhardt, R., Resin Acid Conversion with CYP105A1: An Enzyme with Potential for the Production of Pharmaceutically Relevant Diterpenoids. *ChemBioChem* **2013,** *14*, 467-473.

91. Janocha, S.; Bernhardt, R., Design and characterization of an efficient CYP105A1-based whole-cell biocatalyst for the conversion of resin acid diterpenoids in permeabilized Escherichia coli. *Applied Microbiology and Biotechnology* **2013,** *97*, 7639-7649.

92. Takamatsu, S.; Xu, L.-H.; Fushinobu, S.; Shoun, H.; Komatsu, M.; Cane, D. E.; Ikeda, H., Pentalenic acid is a shunt metabolite in the biosynthesis of the pentalenolactone family of metabolites: hydroxylation of 1-deoxypentalenic acid mediated by CYP105D7 (SAV_7469) of Streptomyces avermitilis. *The Journal of Antibiotics* **2011,** *64*, 65-71.

93. Bleif, S.; Hannemann, F.; Lisurek, M.; von Kries, J. P.; Zapp, J.; Dietzen, M.; Antes, I.; Bernhardt, R., Identification of CYP106A2 as a Regioselective Allylic Bacterial Diterpene Hydroxylase. *ChemBioChem* **2011,** *12*, 576-582.

94. Girhard, M.; Machida, K.; Itoh, M.; Schmid, R. D.; Arisawa, A.; Urlacher, V. B., Regioselective biooxidation of (+)-valencene by recombinant E. coli expressing CYP109B1 from Bacillus subtilis in a two-liquid-phase system. *Microbial Cell Factories* **2009,** *8*, 36-36.

95. von Bühler, C.; Le-Huu, P.; Urlacher, V. B., Cluster Screening: An Effective Approach for Probing the Substrate Space of Uncharacterized Cytochrome P450s. *ChemBioChem* **2013,** *14*, 2189-2198.

96. Ewen, K. M.; Hannemann, F.; Khatri, Y.; Perlova, O.; Kappl, R.; Krug, D.; Hüttermann, J.; Müller, R.; Bernhardt, R., Genome Mining in Sorangium cellulosum So ce56. *Journal of Biological Chemistry* **2009,** *284*, 28590-28598.

97. Schifrin, A.; Litzenburger, M.; Ringle, M.; Ly, T. T. B.; Bernhardt, R., New Sesquiterpene Oxidations with CYP260A1 and CYP264B1 from Sorangium cellulosum So ce56. *ChemBioChem* **2015,** *16*, 2624-2632.

98. Ly, T. T. B.; Schifrin, A.; Nguyen, B. D.; Bernhardt, R., Improvement of a P450-Based Recombinant Escherichia coli Whole-Cell System for the Production of Oxygenated Sesquiterpene Derivatives. *Journal of Agricultural and Food Chemistry* **2017,** *65*, 3891-3899.

99. Rinkel, J.; Litzenburger, M.; Bernhardt, R.; Dickschat, J. S., An Isotopic Labelling Strategy to Study Cytochrome P450 Oxidations of Terpenes. *ChemBioChem* **2018,** *19*, 1498-1501.
